# Globally deployed COVID-19 fever screening devices using infrared thermographs consistently normalize high readings to afebrile range

**DOI:** 10.1117/1.JBO.26.4.043009

**Published:** 2021-03-13

**Authors:** Conor Healy, Zachary Segal, Chris Hinnerichs, Ethan Ace, Derek Ward, John Honovich

**Affiliations:** a IPVM, Bethlehem, Pennsylvania, United States; b Infrared Thermography for Febrile Screening in Public Health – ISBN-13: 978-3659263194

**Keywords:** thermography, febrility, screening, NCIT, IRT, pyrometry

## Abstract

**Significance:** The need for regulatory review of infrared thermographs (IRTs) used on humans was removed in response to the unique circumstances of the SARS-CoV-2 pandemic (a.k.a., COVID-19). The market for these devices has since expanded considerably. This evaluation of IRT performance may have significant implications for febrility screening worldwide.

**Aim:** Perform controlled nonhuman trials of IRT devices to identify and quantify deviations in the human temperature range.

**Approach:** We compared IRT readings of a temperature-controlled non-human subject with one FDA-cleared IRT and one FDA-cleared handheld NCIT. In individual trials for each device, the subject was measured between 35°C and 40°C at 0.25°C increments.

**Results:** The IRT device measurements were consistently normalized around the human mean (∼37°C). Temperatures were decremented as they approached the febrile range, and systematically reported as normal across all seven devices. This effect does not appear to be explained by a fixed offset or any known approach to estimating body temperature, or by random error.

**Conclusion:** The IRTs in this study generated human temperature measurements that were systematically biased to the mean human temperature. Given that these devices are utilized for sentinel detection of possible infectious disease transmission, and are now globally employed, the implications for reduced detection of febrility are a widespread false sense of security. This vulnerability must be considered with respect to facility access control, clinical applications, and travel screening in the context of the ongoing COVID-19 pandemic response.

## 1 Introduction

The COVID-19 pandemic prompted rapid mobilization of temperature screening devices using infrared thermographs (IRTs) to deployment in airports, schools, and workplaces. IRTs are known for their potential application to noncontact mass fever-screening, and research has demonstrated that IRTs can provide sufficient accuracy for human applications.^1^ Nevertheless, it is generally understood (in the literature) that controlled conditions are required if IRTs are to be relied upon for human febrility screening.^2^ Performance is negatively affected by such factors as natural sunlight, ambient temperature, air circulation, insufficient device warm-up time, and incorrect calibration and/or calibration without an external reference device, or calibrated black body (BB); incorrect preparation of the subject also impacts performance. Operational guidelines for IRT-based screening have been published by the ISO/IEC;^3^^,^^4^ however, these controlled conditions are difficult to replicate as applied in industry. Thus, prior to the COVID-19 global pandemic declaration by the World Health Organization (WHO) on March 11, 2020, IRTs were only utilized in a few foreign international airports for mass fever screening.

In the United States, the FDA regulates IRTs marketed for use on humans as medical devices. The FDA requires that newly marketed medical devices, such as IRTs, complete a process to demonstrate safety and effectiveness; this is known as a 510(k) clearance. However, in April 2020, the FDA announced that the agency would not object to unvetted devices insofar as they “do not create an undue risk.” This position was taken to minimize regulatory oversight, which might constrain the supply of temperature screening solutions.^5^

Following the FDA’s announcement, a surge of unvetted IRT-based human temperature screening products went directly to market. More than 214 companies now market such medical devices, of which a significant majority first entered the market in 2020 during the COVID-19 pandemic without any previous IRT or medical device product lines, according to independent industry research by IPVM.^6^ New, unvetted devices are often marketed for use under conditions that violate ISO/IEC guidelines including, but not limited to, uncontrolled environmental conditions, improper preparation of subjects, and lack of calibration devices.^7^^,^^8^ Our study contributes importantly as a surrogate for the typical FDA review process insofar as these new IRT models diverge from the typical performance expectations of thermographic devices. IPVM is an independent publication funded by membership fees that provides access to research findings related to various surveillance and access control device components and functions. This study was conducted at the $12,000\;{\mathrm{ft}}^{2}$ IPVM laboratory facility in Bethlehem, Pennsylvania and extends upon extensive independent testing by IPVM of commercially available IRTs introduced during the COVID-19 pandemic.^6^

A recently published study which evaluated the accuracy of IRTs against a human subject’s oral reference temperature found relatively good agreement (correlation of 0.75) in a controlled setting following all consensus guidelines.^9^ A recent meta-analysis of 19 studies related to IRTs and noncontact infrared thermometers (NCITs) against a reference standard thermometer found a low positive predictive value at the early stage of a pandemic, but the negative predictive value remained high. For NCITs using the forehead, the pooled sensitivity was 0.808 (95% CI 0.656 to 0.903) and specificity was 0.920 (95% CI 0.769 to 0.975). Regarding IRTs, the pooled sensitivity was 0.818 (95% CI 0.758 to 0.866) and specificity was 0.923 (95% CI 0.823 to 0.969).^10^ In contrast, our study situated the devices in a controlled laboratory setting to establish performance against a non-human subject with precise temperature settings, further controlling performance conditions. Our goal was not to assess accuracy against a human target, but rather to detect any systematic adjustments or other deviations in the readings. As such, our study complements the human target studies using laboratory conditions and repeat measurements for each device. This research provides the first known laboratory-controlled evaluation of seven new IRTs versus two 510(k)-cleared IRTs to quantify the direction and magnitude of any proportional bias evident in the measurements.

If any nonrandom deviations in measurements from actual target temperature are to be expected, they are not disclosed in product registration materials, manuals, data sheets, specifications, or marketing materials. Given that any onboard compensating algorithms are not open source, establishing the magnitude and direction of adjustment is imperative to evaluating a new entrant’s validity for screening. Although secondary screening may occur in IRT deployments, typically with a handheld NCIT, it is only performed when the IRT reports elevated temperatures. In summary, the importance of accurate screening is particularly relevant to the natural history of COVID-19 given that the incubation period is followed by an infectious prodrome period, during which time individuals may be asymptomatic.^11^ An elevated temperature may be as low as 37.5°C. Any normalization of IRT readings to the human mean temperature reduces the product’s utility as a screening device, even where secondary screening is available.

### 1.1 Sensors and Target Selection in New IRTs

Newly released IRTs commonly use IR sensors with much lower resolution than FDA-cleared IRTs. For example, Heimann sensors are a widely used component “temperature tablet” IRTs, which are available in resolutions ranging from 16×16 to 80×64  pixels; sensors with a resolution of 32×32  pixels or 1024 pixels in total, are the most common.^12^ By comparison, the FDA-cleared FLIR E54 (a control device in this study) uses a sensor of 320×240  pixels or 75 times as many pixels at 76,800 pixels. Per manufacturer specifications, the distance of the subject from the IRT may be as far as 1 m (∼3  ft). The low resolution and long screening distance may yield potentially unreliable readings as they may not capture the medial canthus.

The new devices also differ from conventional thermography with respect to target selection. Conventionally, IRTs target the medial canthus (a.k.a., inner canthus) for human temperature measurement as the evidence suggests this area most closely approximates the surface temperature best correlated to core temperature. In contrast, the new devices typically target either the warmest part on the entire face irrespective of location or the forehead region. Notably, the studied devices in this article apply a compensating algorithm only upon detection of a human head, suggesting that whatever purpose the algorithm is intended to serve is human-specific.

### 1.2 Approach to Estimating Human Core Body Temperature

Human temperature varies depending on the part of the body that is measured. The range of human temperatures is usually defined in terms of core body temperature or oral temperature in clinical settings. Core temperature is usually estimated with oral temperature, tympanic temperature (ear), or surface temperature (skin temperature), among others. Devices measuring oral, tympanic, or surface temperatures for diagnostic purposes either report raw measurements or report an estimate of core body temperature computed with an on-board algorithm. The relationship between the accuracy of core body temperature estimates may vary depending on the location measured and the device design for computing an estimate.

All IRTs measure the surface temperature of the face, with edge-case exceptions. Some devices utilize the surface temperature input to provide a reading that estimates body temperature; however, the conversions vary by device. The approach of recently introduced IRTs is poorly understood because the manufacturer specifications do not include the conversion algorithm, and to our knowledge, no independent assessment has been published to date. Nevertheless, it is well established that surface temperature is lower than core body temperature and lower than oral temperature.^13^^–^^15^ Therefore, the IRT estimates of core body temperature would be expected to be higher than the surface temperature measured.

## 2 Methods

Seven IRT devices were selected for testing according to the following criteria: (1) the device was introduced in 2020; (2) the device went directly to market without FDA 510(k) regulatory review; (3) the device is likely to be used or was known to be used. The third criterion was assessed informally and, depending on the device, as discussed in Sec. 2.1. Lacking verifiable sales data, this evaluation was based on marketing claims or, in some cases, evidence of use by well-known institutions. By selecting seven devices, we sought to obtain a more comprehensive dataset while reducing the possibility that any single device was faulty, or otherwise unrepresentative of the performance of its class. Two control devices were selected according to the criteria that they are 510(k)-cleared. (Table 1)

**Table 1:** Devices stated accuracy and emissivity by type.

IRTs, NCIT, and blackbodies used	Type	Stated accuracy	Stated emissivity
Extech IR200: noncontact forehead infrared thermometer	NCIT	±0.3°C	NA
FLIR E54-EST thermal screening solutions	IRT	±0.3°C	NA
TVT TD-E2128-TM face recognition terminal	IRT	±0.3°C	NA
Bems temperature terminal	IRT	Not stated	NA
Dahua DH-TPC-BF5421-T thermal hybrid network camera	IRT	±0.33°C	NA
ZKTeco SF1008+ body temperature + mask detection access control reader	IRT	±0.33°C	NA
Certify SnapXT Pro	IRT	±0.3°C	NA
Hikvision DS-2TD2636B-13/P temperature screening thermographic camera	IRT	±0.5°C	NA
Meridian clear 2 temperature screening Kiosk	IRT	±0.5°C	NA
Dahua JQ-D70Z blackbody	BB	±0.2°C	0.97
Sunell SN-TH01 portable blackbody	BB	±0.2°C	0.97

Each of the seven test devices was evaluated individually in separate trials. In each trial, the subject was measured at all 0.25°C increments between 35°C and 40°C to mimic low, normal, and elevated human surface temperatures, yielding a total of 21 measurements per tested device. For each measurement, in each trial, measurements were also recorded by the two control devices to ensure that if round-to-round variation in conditions had an impact, it would be observed; to verify the temperature setting of the subject’s BB device with a scanner vetted for accuracy; and to have a vetted comparison for all readings by tested IRTs. Our testing procedure was designed to conform FDA screening procedures and manufacturer specifications, IRT thermo-science literature, and international standards and consensus guidelines.^1^^,^^3^^,^^16^

### 2.1 Devices

The devices selected included five “temperature tablets” (a.k.a., temperature kiosks, temperature terminals) and two “bullet camera” IRTs. (Table 1) The temperature tablets were as follows: Bems Temperature Terminal, TVT TD-E2128-TM, Certify SnapXT Pro, Meridian Clear 2, and the ZKTeco 8” SF1008+. The bullet camera IRTs were the Dahua DH-TPC-BF5421-T and the Hikvision DS-2TD2636B-13/P.

All fit the first criterion in that they were introduced in 2020. None have undergone the 510(k) clearance process, our second criterion. Regarding the third criterion, Hikvision and Dahua are the largest and second largest video surveillance manufacturers in the world, respectively. For both, there is evidence of worldwide sales of IRT temperature screening products.^17^^–^^19^ In marketing materials, Certify claims to be utilized in 75% of NFL stadiums.^20^ Meridian states they are used by numerous school districts^21^ and has been purchased by the U.S. Department of Veterans Affairs, which administers hospitals across the United States.^22^ Bems and TVT supply temperature tablets for rebranding to multiple companies worldwide.^23^^,^^24^ The ZKTeco device was selected because it is one of the world’s largest access control and biometrics manufacturers, with clients including Walmart and Carrefour.^25^

The two control devices were the Extech IR200, a handheld NCIT, and the FLIR E54 EST, an IRT. Both were FDA 510(k)-cleared.

### 2.2 Approach

As stated previously in Sec. 1, IRT measurement capability is negatively affected by various factors, namely poor environmental conditions and improper subject preparation. The laboratory setup was designed to ensure our observations were representative of true device performance; that is, with minimal impact on performance by interfering factors.

Specifically, we sought to replicate conditions under which IRTs have been tested in literature^9^ and expected to perform normally, including following FDA guidelines, the IEC 80601 standards, and manufacturer specifications. Testing was conducted in a windowless indoor laboratory with concrete walls. The area was temperature controlled, and ambient temperature and humidity were verified for each trial with an Extech RH520A Humidity+ Temperature Chart Recorder with Detachable Probe (FLIR Systems, Oregon, United States of America). All devices, inclusive of all IRTs, BBs, and NCITs were turned on to warm-up 30 min prior to the start of testing (in accordance with manufacturer guidelines). To minimize airflow, we ensured there were no open doors, or active air-conditioning, heating, or fans. IRT optics were checked for lens contamination (e.g., dust, debris, and fingerprints) that could skew readings. If devices had calibration settings, they were calibrated per manufacturer guidelines. Three of the IRT devices, the Dahua, Hikvision, and Meridian, and the Extech NCIT had methods for manual calibration, which was performed with the Dahua BB reference device set at 37°C. While the human body core temperature is roughly 1°C above forehead temperature, calibration at 37°C was appropriate to avoid introducing bias. In addition, a fixed offset will not impact trends, variance, or correlation. The Meridian was given a fixed offset of ±0.2°C, the Hikvision and Dahua IRTs were each given an offset of −1.6°C; these offsets calibrated the IRTs such that when the reference device was set at 37°C, the IRT read 37°C. Finally, testing was conducted with a low reflective background, mounted (fixed) and on a horizontal plane directly in front of the subject.

**Fig. 1:**
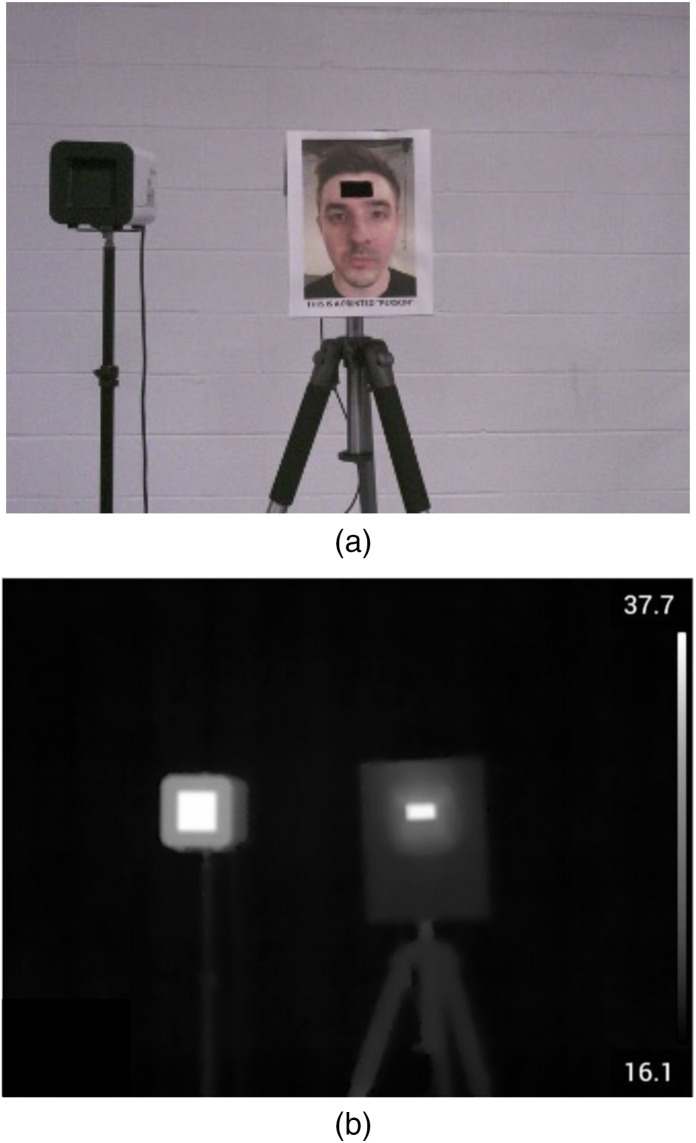
Testing configuration for simulated human thermographic reading. (a) Regular image. (b). Thermal image. Subject was mounted at 168 cm from the floor. Manufacturer specifications were followed in the placement of the IRTs relative to the subject. All tablets were mounted at a height of 155 cm and placed at a distance of 50 cm from the subject. The FLIR IRT was mounted at a height of 157.5 cm and placed 70 cm from the subject.

**Fig. 2:**
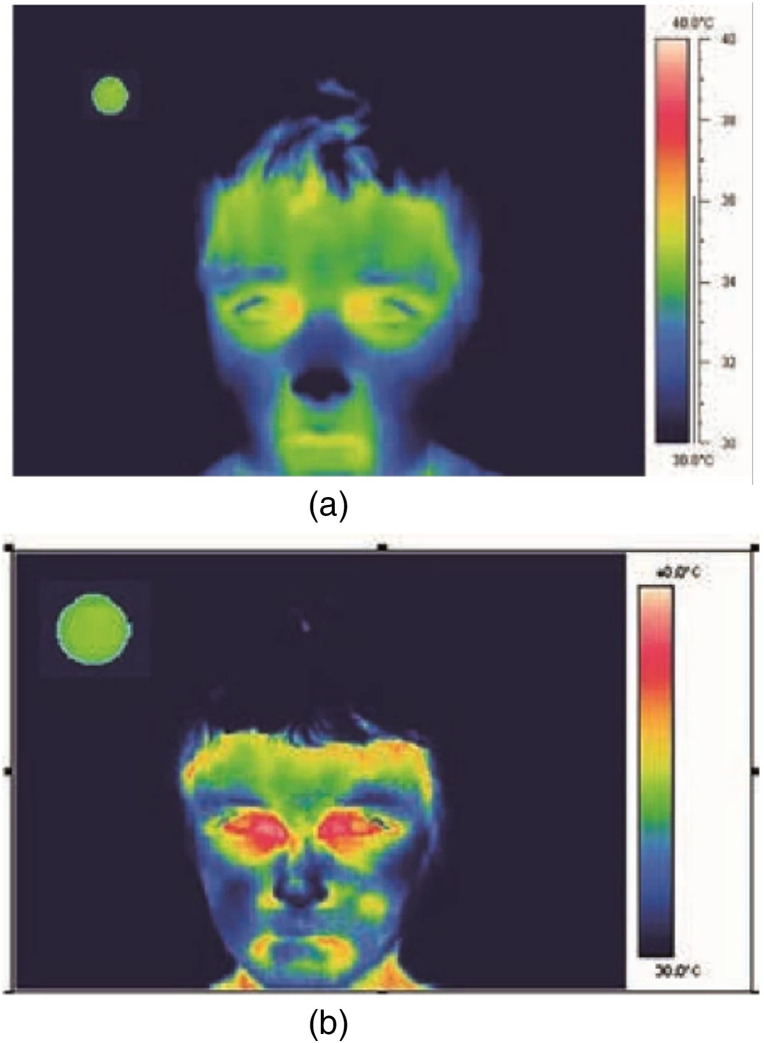
IRT image of (a) male with normal temperature and (b) male with elevated temperature from IEC 80601.^3^

**Fig. 3:**
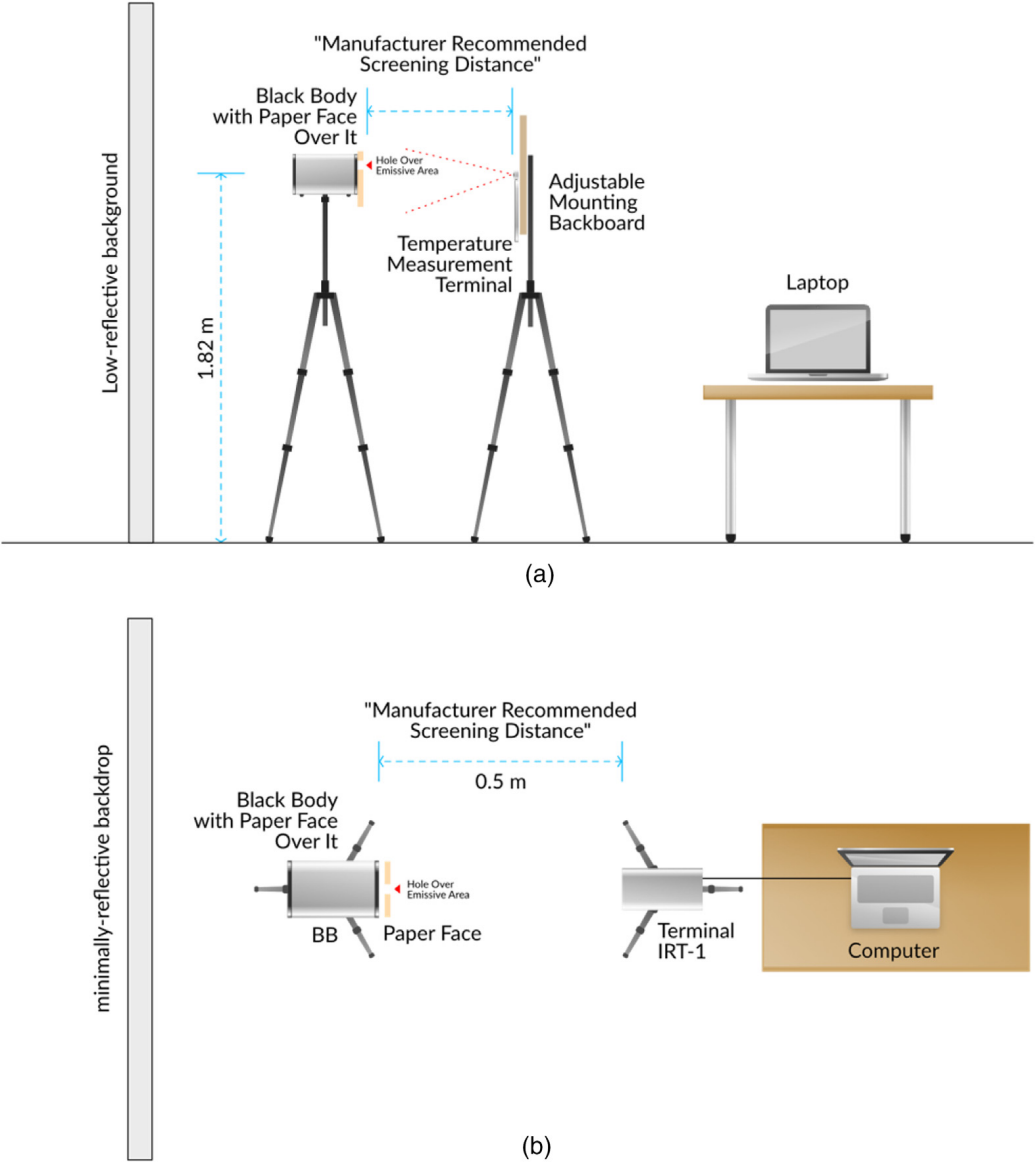
Testing environment. (a) Side view. (b) Top view.

Since IEC, FDA, and manufacturer guidelines are intended for use with human subjects, our procedure differed by employing a non-human subject. We used a human face printed on generic 9.5  in×11  in. white printer paper attached to a paperboard to ensure a flat surface (all test devices only display temperature readings upon detection of a human face). An opening in the paper face and paperboard of 5 cm width by 2.5 cm height was made over the paper face’s forehead. We used a BB of known emissivity and accuracy as the measurement target, the Sunell SN-TH01. The paperboard, with the printed face, was placed over the BB device such that its radiant area protruded from the cutout to allow direct measurement by the IRTs/NCITs [Figs. 1(a) and 1(b)]. This setup, the BB, and printed face are hereafter referred to as the “subject.” All IRT devices measured the subject’s radiant area, which was the highest thermal yield displayed in the field of view. The exposed surface area of the subject was $12.5\;{\mathrm{cm}}^{2}$, providing a large target with uniform temperature and high emissivity, conditions favorable to IRT performance [Figs. 1(a) and 1(b)]. By comparison, the surface area of the human face from which febrile temperatures can be observed is considerably smaller and less uniform, ensuring that the test process was conservative (Fig. 2). This setup is thought to be more favorable to IRT performance than real-world testing environments, because the subject is uniformly heated, still, and unobstructed, and various environmental factors likely to affect performance were eliminated [Figs. 3(a) and 3(b)].

**Fig. 4:**
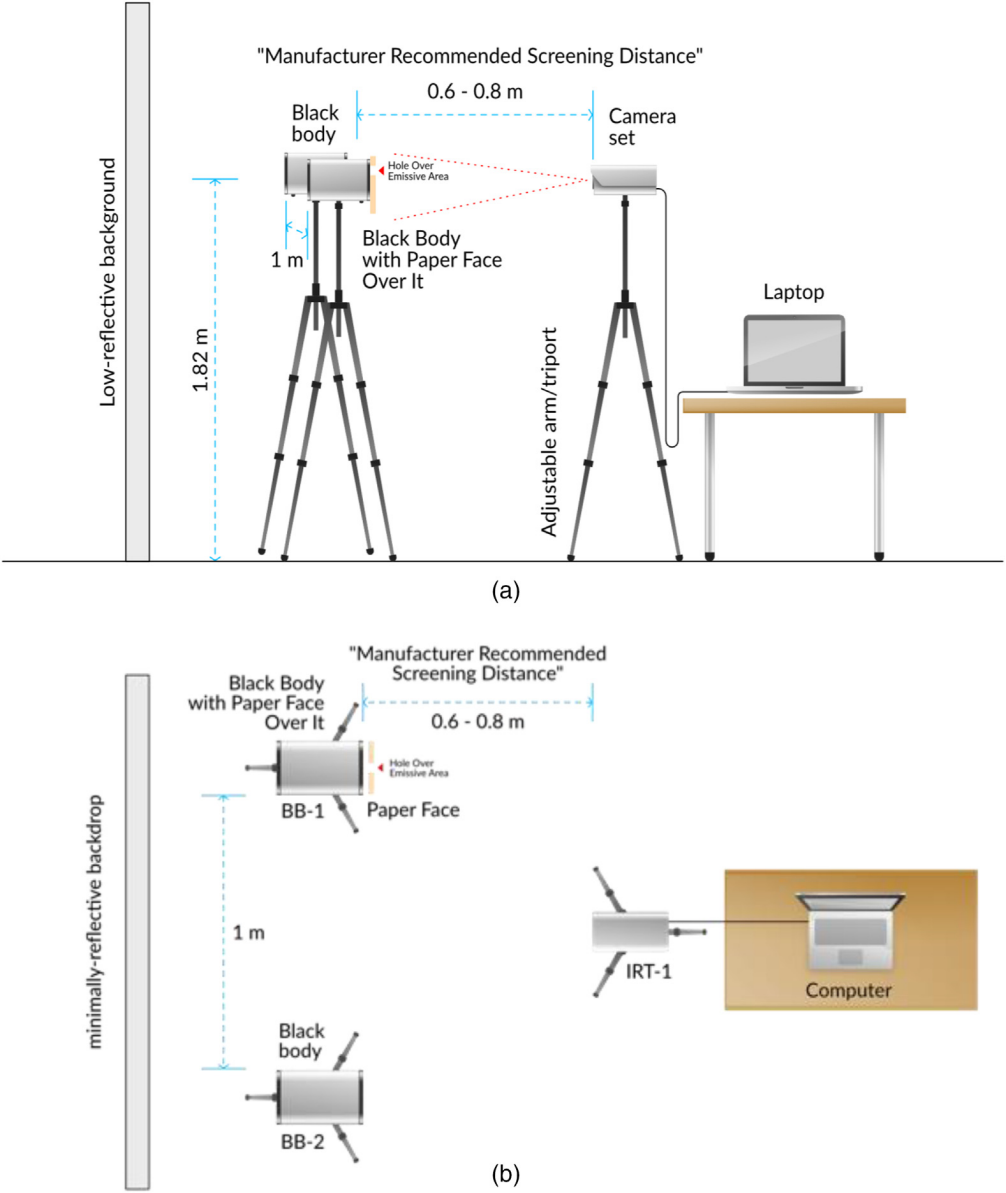
Testing environment for devices requiring BB calibration with subject and reference device placed side-by-side. (a) Side view. (b) Top view.

For trials of the Dahua and Hikvision bullet IRTs, we used an accompanying external reference device per manufacturer instructions. This was the Dahua JQ-D70Z BB, selected for its compatibility with these products. The reference device was placed 1 m apart from the subject to avoid interference, and each was 3 m away from the bullet IRT being tested. The reference device was placed at a height of 1.8 m, the subject was placed at a height of 2 m, and the bullet IRT was also placed at a height of 2 m. Both the subject and reference device were within the camera’s field of view. We calibrated the bullet IRTs with the reference device after the 30-min warm-up. The FLIR IRT was at a height of 157.5 cm and 70 cm from the subject. The reference device was not in the FLIR IRT’s field of view [Figs. 4(a) and 4(b)].

The readings for each of the seven tested devices were recorded between 35°C and 40°C at 0.25°C increments, yielding 21 readings per device. For each increment, we took a measurement with the IRT being tested, one with the FLIR E54 EST, one with the Extech NCIT in surface mode, and two with the Extech NCIT in body mode. Two measurements were taken with the NCIT in body mode to confirm that the subject’s BB was measured, not the paper; these results were averaged for the subsequent analyses.

## 3 Results

Descriptive statistics were computed for all devices, including mean output, range, and variance (Table 2). We also used linear regression to associate the relationship between the device reading and subject’s temperature setting, as well as the discrepancy between tested and control devices. With the sole exception of the Meridian IRT (see Table 2 for logarithmic regression), neither logarithmic nor exponential regression yielded an improvement in fit, as measured by an r-squared of more than 0.02, indicating linear regression is an appropriate estimation of the relationship between device readings and subject temperature setting.

**Table 2:** Deviation from control temperature reading.

Device	Range (°C)	Variance °C	Mean °C	Device versus subject temp.		Difference between device and subject versus subject temp.	
				Adjusted R-squared	Regression coefficient	Adjusted R-squared	Regression coefficient
Control devices							
FLIR IRT	35 to 40	2.4	37.5	0.99	1	0	−0.001
Extech surface NCIT	35.05 to 40.05	2.4	37.5	0.99	1	0	−0.001
Extech body NCIT	36.7 to 40.2	1.3	38.2	0.98	0.78	0.8	−0.22
Tested devices							
Hikvision Bullet IRT^a^	35.6 to 39.6	1.6	37.4	0.99	0.8	0.88	−0.19
Dahua Bullet IRT^b^	35.4 to 39.4	1.4	37.3	0.99	0.77	0.95	−0.23
Meridian IRT^c^	36.9 to 39.8	0.95	37.8	0.91	0.6	0.82 (.91 log)^d^	−0.4
ZKTeco IRT	36.3 to 39.0	0.85	37.3	0.94	0.58	0.89	−0.42
Certify IRT	36.4 to 39.0	0.72	37.3	0.93	0.53	0.91	−0.47
TVT IRT	36.6 to 37.8	0.16	36.9	0.7	0.22	0.97	−0.78
Bems IRT	36.5 to 36.8	0.005	36.7	0.001	0	0.99	−1

a Calibrated with BB reference at 37°C with a fixed offset of −1.6°C as per manufacturer recommendations.

b Calibrated with BB reference at 37°C with a fixed offset of −1.6°C as per manufacturer recommendations.

c Calibrated with BB reference at 37°C with a fixed offset of +0.2°C as per manufacturer recommendations.

d An adjusted R-squared of 0.91 using logarithmic regression. This was the only device where logarithmic regression improved adjusted r-squared by 0.02 or more.

### 3.1 Analysis of 510(k)-Cleared Control Devices

The control devices were consistent and accurate, recording small differences from the subject’s actual BB temperature setting over all 21 measurements from 35.0°C to 40.0°C and across all seven rounds of testing, indicating that nothing about ambient conditions or the testing environment produced an inaccurate reading (Table 2). The two Extech NCIT surface mode readings never differed from each other by more than 0.2°C, and all readings were within 0.1°C of the subject. The FLIR performed similarly, with readings never varying by more than 0.2°C and all readings within 0.15°C of the subject. The Extech NCIT body mode averages were consistent but differed more from the subject setting than in surface mode. This is to be expected because the device uses an algorithm to estimate body temperature from surface temperature. In body mode, the averaged Extech NCIT varied by 0.45°C or less. These results suggest that the test environment was conducive to accurate IRT and NCIT performance.

### 3.2 Analysis of Tested IRTs

**Fig. 5:**
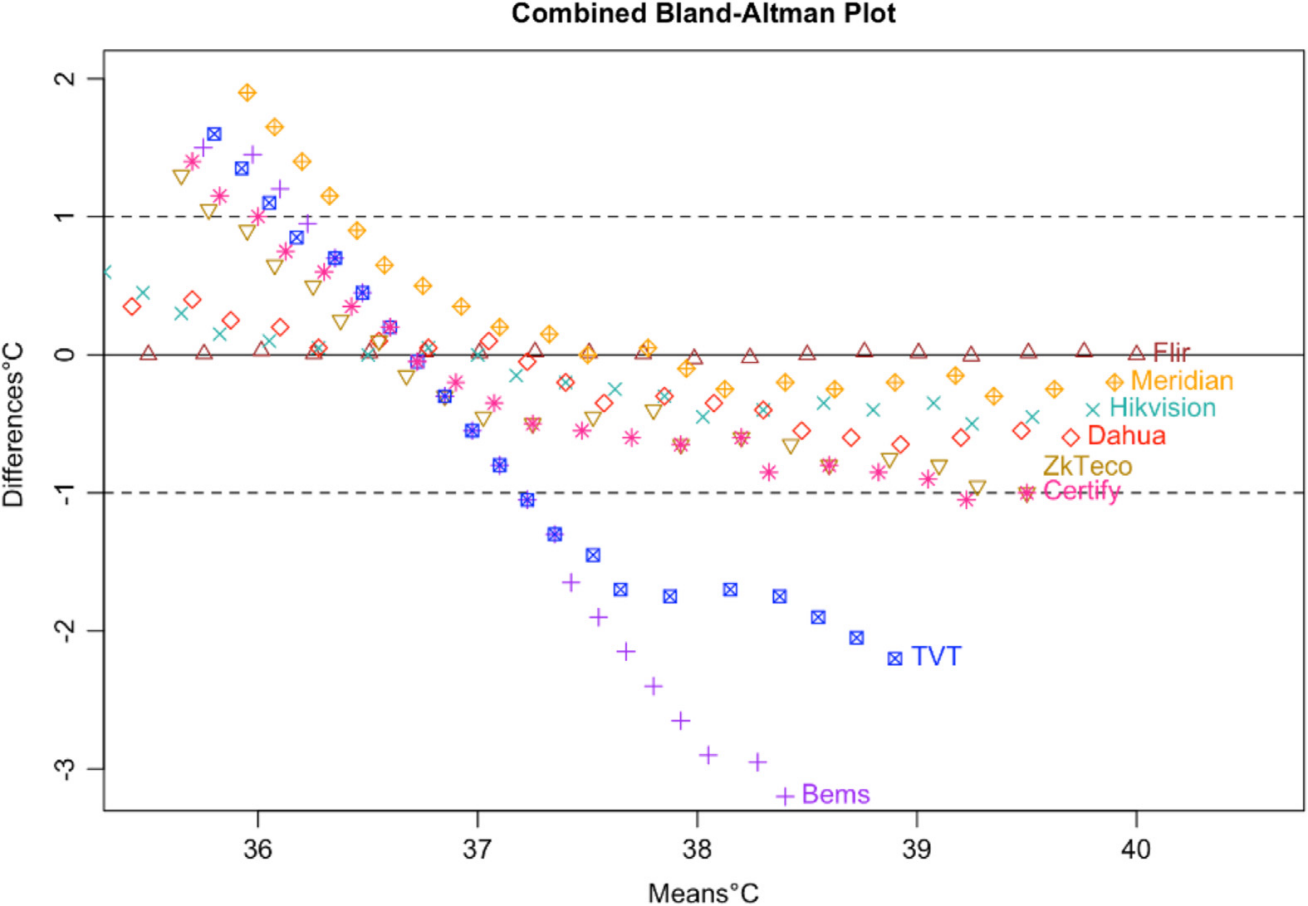
Bland–Altman plot of seven tested devices.

In contrast to the FLIR and Extech control devices, all seven tested IRTs recorded temperatures that deviated from the subject’s temperature setting, systematically reducing temperatures above 37.0°C and increasing those lower than 36.0°C. Specifically, the devices tended to record temperature deviations toward a human mean of 36.0°C to 37.0°C, with more marked reductions found as temperature increased above the mean (Fig. 5). In contrast, the 510(k)-cleared control devices demonstrated near-perfect correlations with the subject; the variance in readings was uniformly dispersed across the range of temperatures measured.

**Fig. 6:**
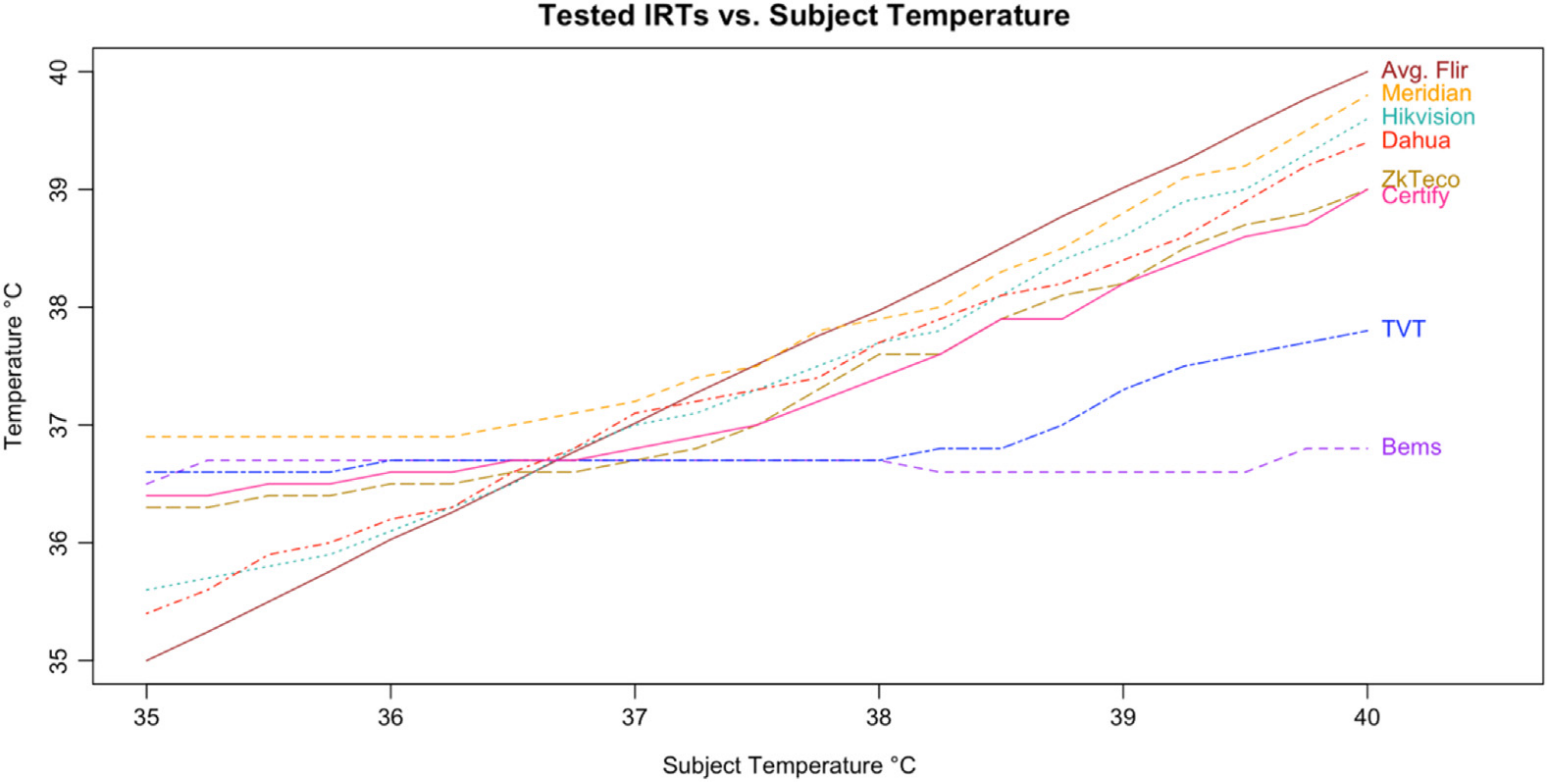
Recorded readings for tested IRT reading versus subject.

Following the calculation of descriptive statistics as described in Table 2, we constructed Bland-Altman plots to visually inspect the limits of agreement between the tested devices and the subject (Fig. 6). We found strong evidence of variable proportional bias in the tested devices but no such bias in the control devices. While the FLIR had nearly zero bias, which appeared to be dispersed randomly, tested devices deviated from the subject and had biases that were proportional to the mean temperature.

**Fig. 7:**
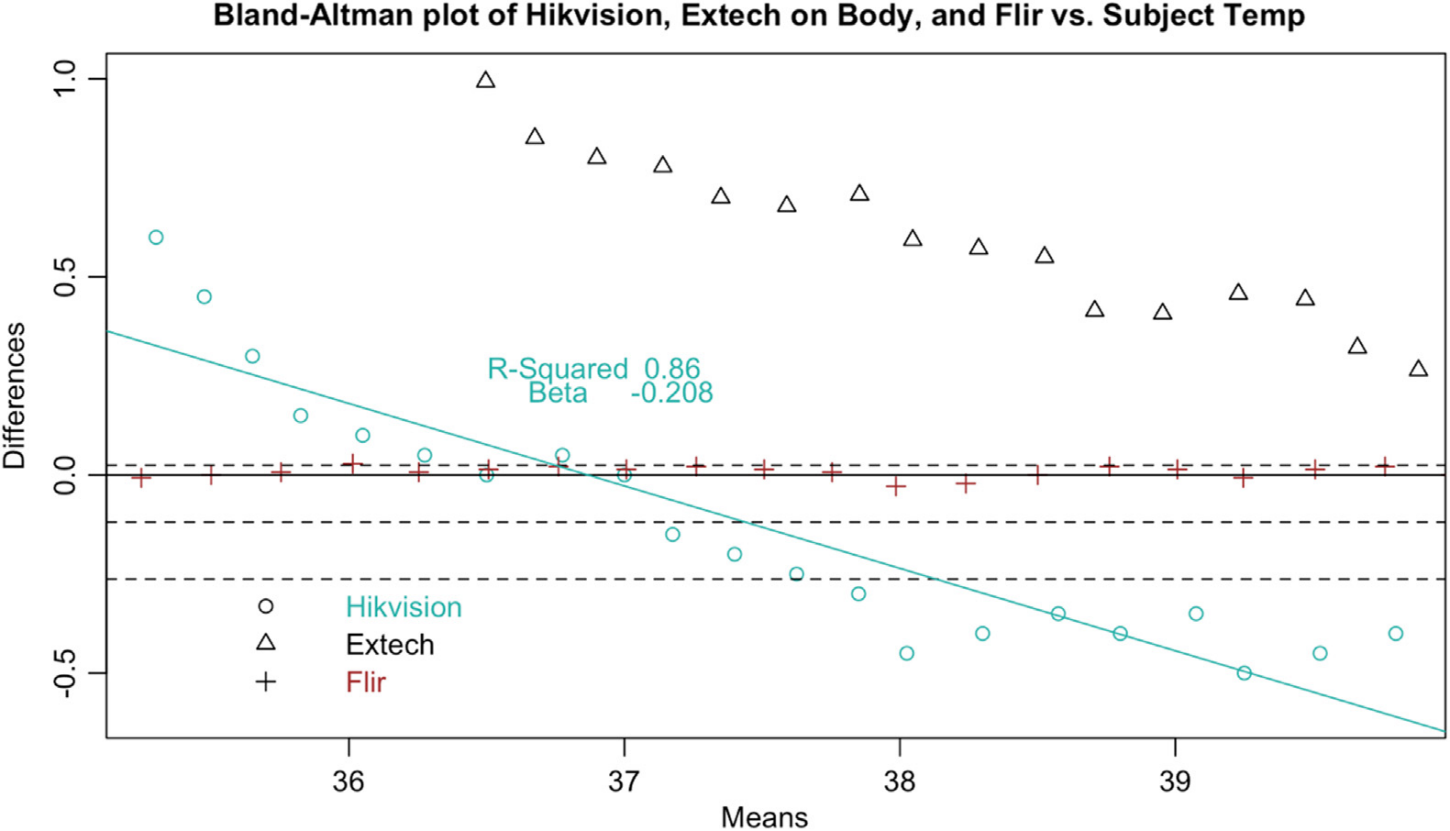
Bland–Altman plot of Hikvision DS-2TD2636B-13/P Results.

**Fig. 8:**
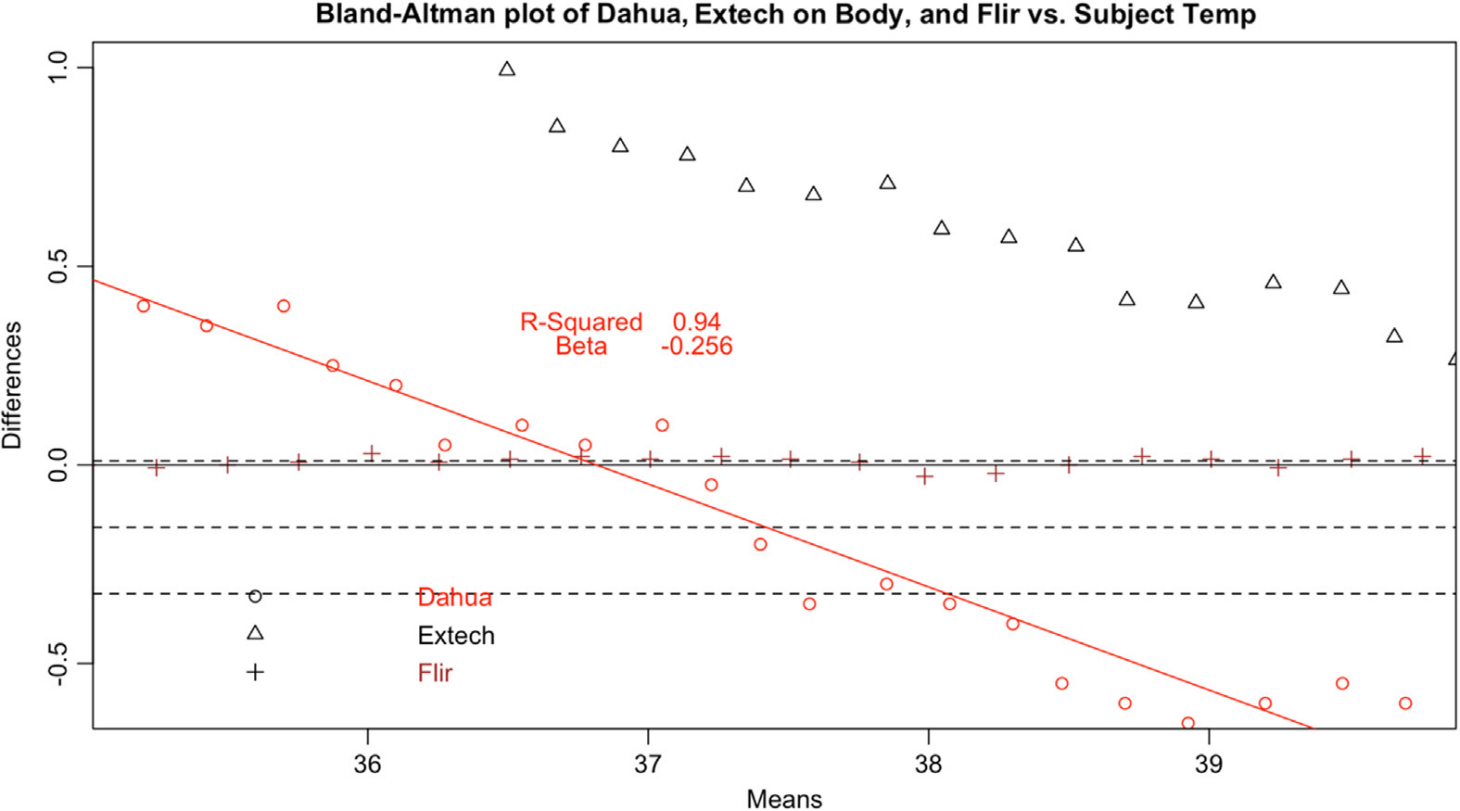
Bland–Altman plot of Dahua DH-TPC-BF5421-T.

**Fig. 9:**
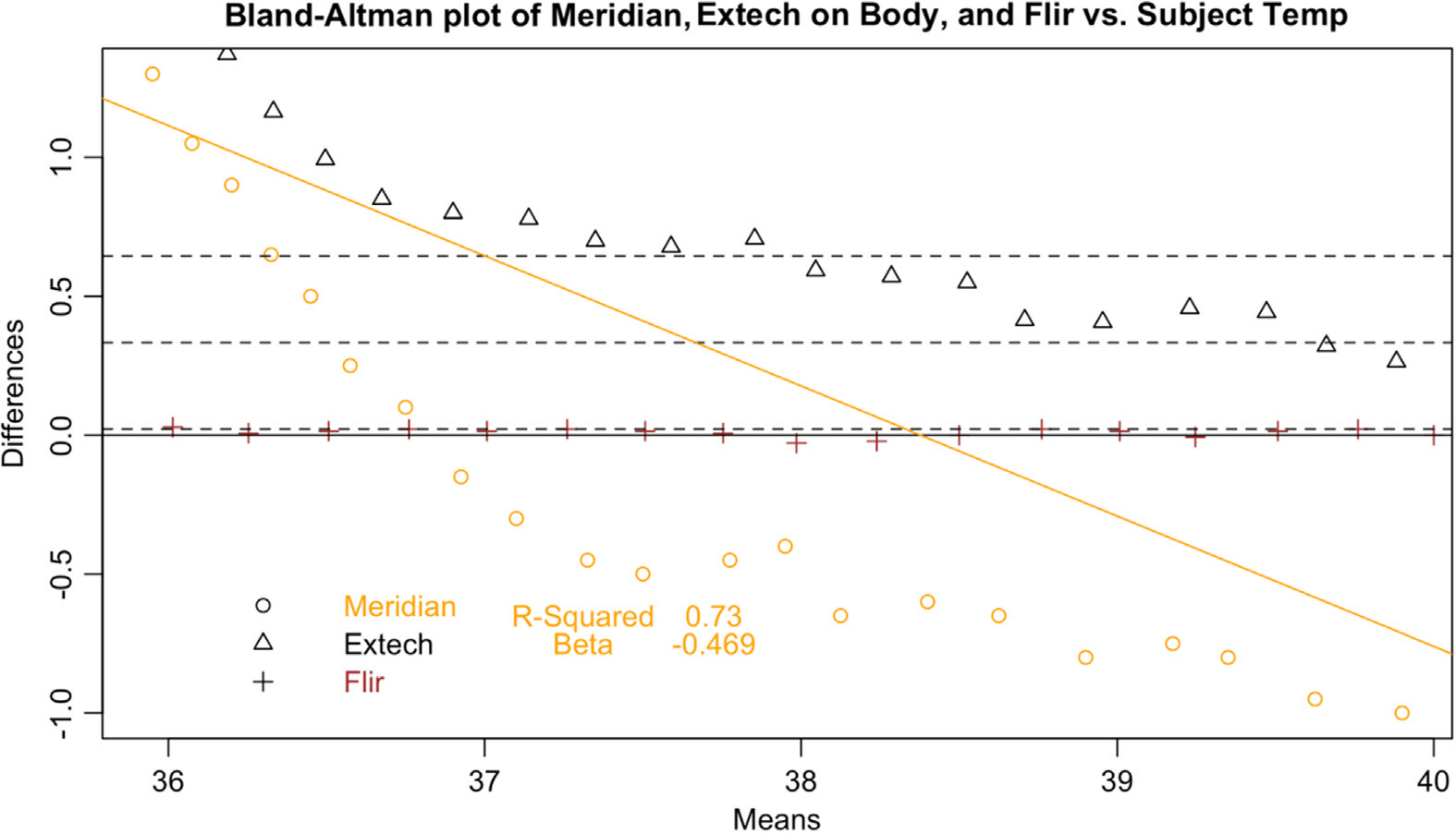
Bland–Altman plot of Meridian.

**Fig. 10:**
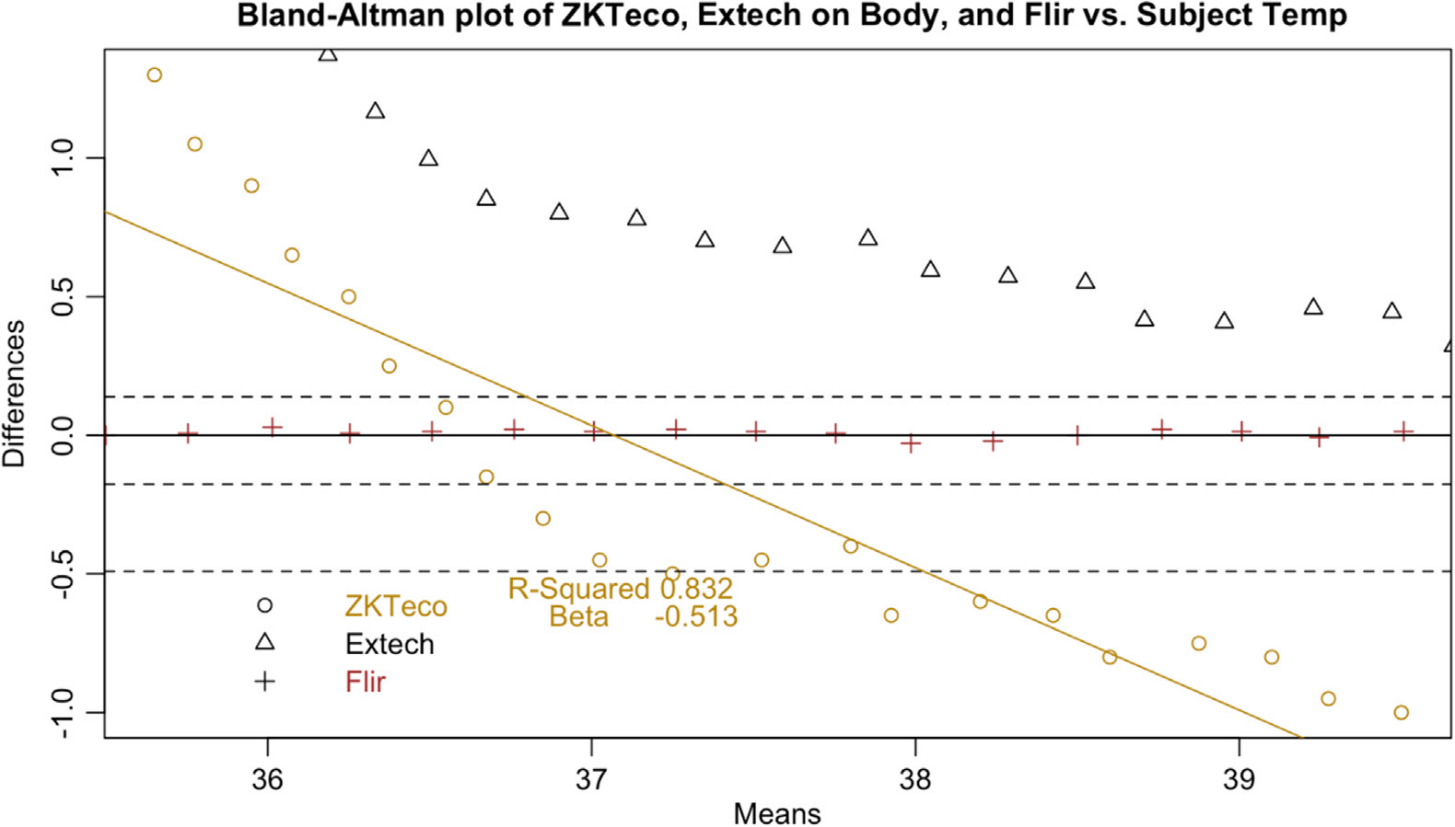
Bland–Altman plot of ZKTeco.

**Fig. 11:**
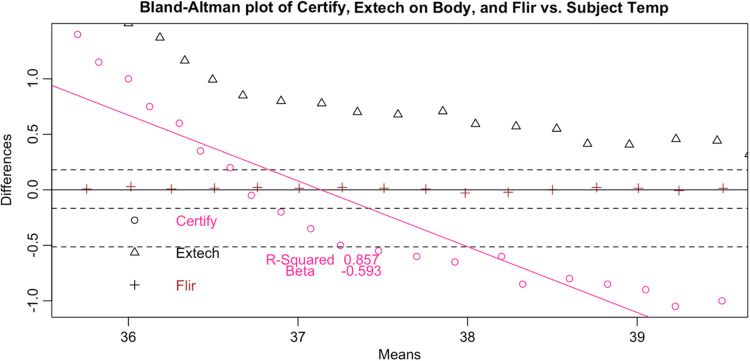
Bland–Altman plot of Certify.

**Fig. 12:**
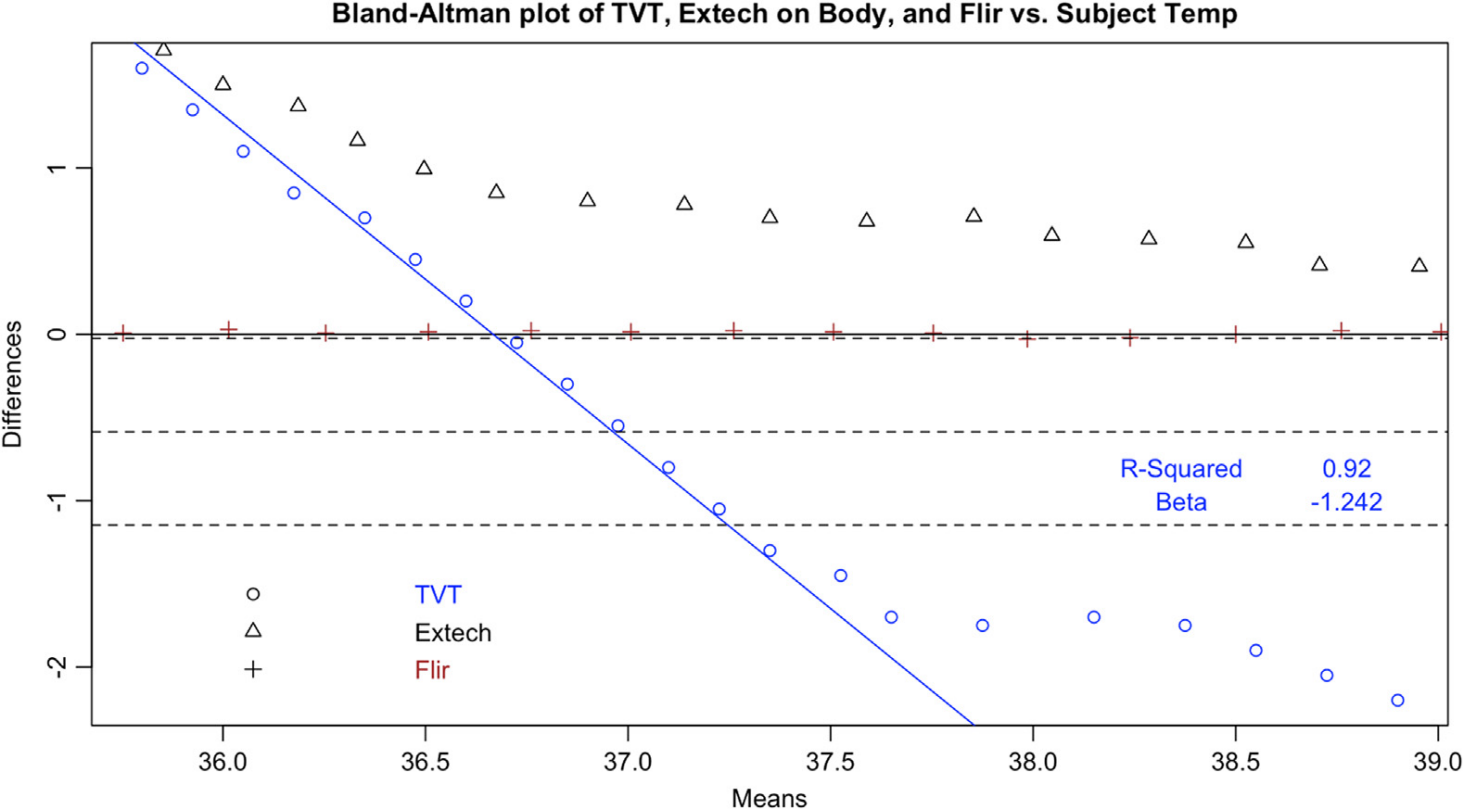
Bland–Altman plot of TVT.

**Fig. 13:**
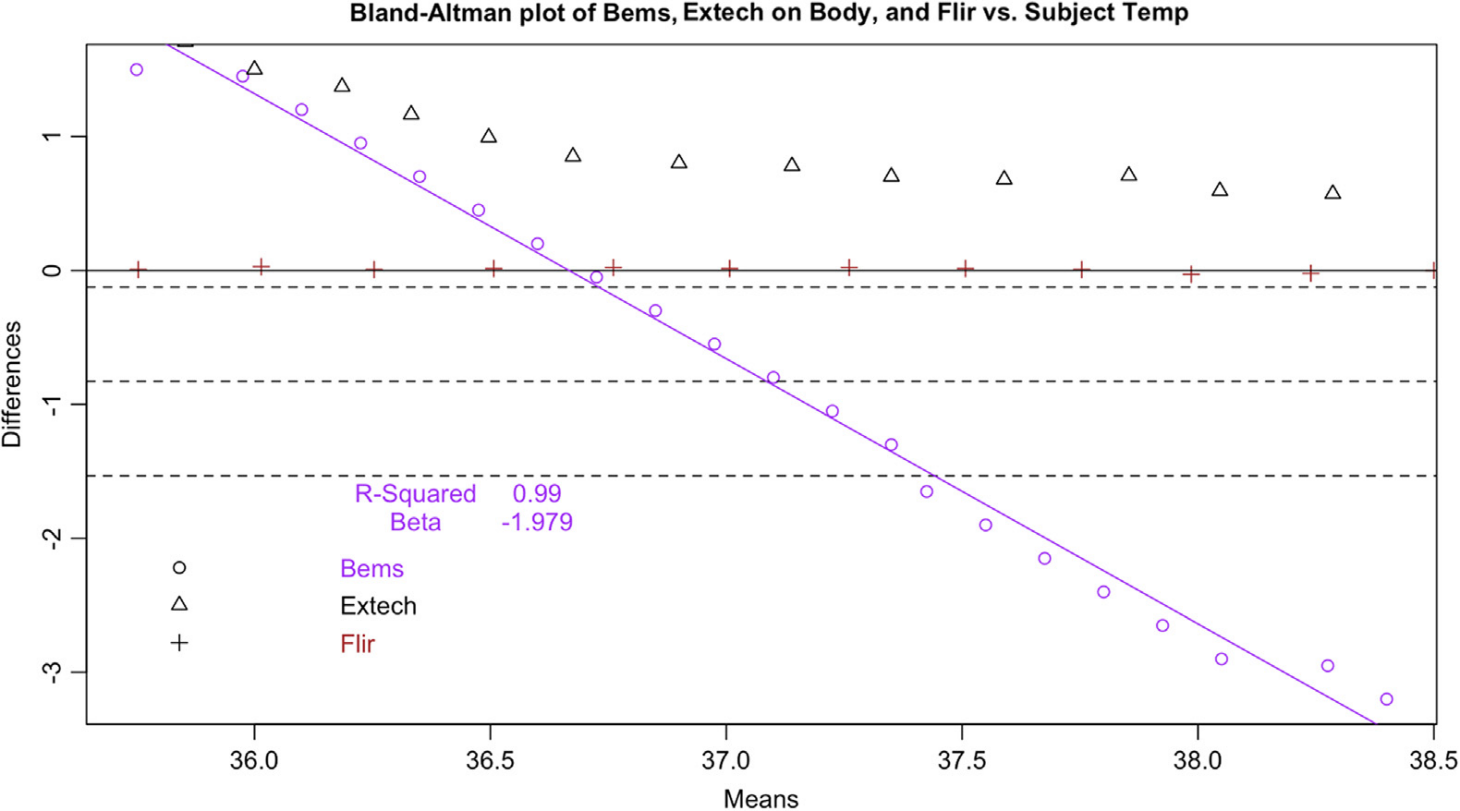
Bland–Altman plot of Bems.

Specifically, we found that the tested IRTs each had proportional biases that decreased upon approach from hypothermic to the human mean, elevating the temperature. As temperature increased past the human mean into the febrile range, the bias then changed direction from positive to negative, thereby reducing the temperature. While the degree of bias varied with each specific device, it was clearly present and proportional in the tested devices. For comparison, the 510(k)-cleared FLIR demonstrated virtually zero bias across the readings whereas the Bems demonstrated significant bias (−0.829), nearly always recording a near-normal temperature regardless of the subject’s temperature setting (Fig. 5).

In addition, all tested IRTs were not as strongly correlated with subject temperature as would be expected for a device that is intended to accurately measure febrility. None had a regression coefficient above 0.8 with subject temperature (a coefficient of 1 would indicate a perfect correlation, and a slope of 1 on a graph, as in the dotted line in Fig. 6); and only the Bullet IRTs regression coefficients were above 0.6 (Figs. 6–13). This shows changes in surface temperature do not lead to equivalent increases in IRT output. An accurate device free of bias or smoothing would generate a regression coefficient closer to 1, as seen with FLIR and Extech (Table 2). In other words, one might expect a consistent offset (e.g., a straight line with slope of 1) but not a systematic and variable norming to the human mean.

**Fig. 14:**
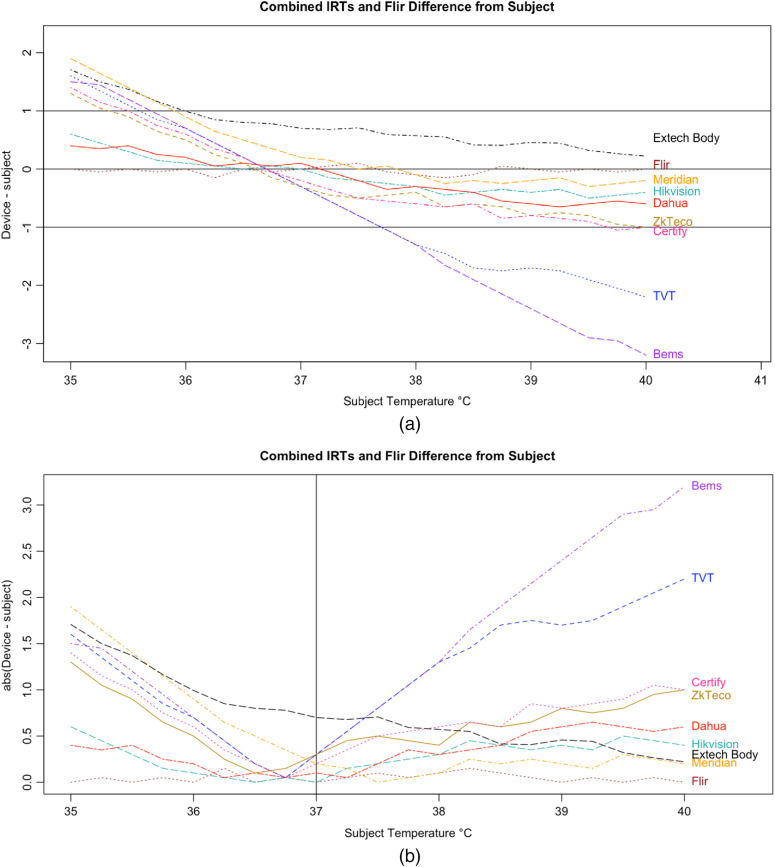
Disparity between control (FLIR) and tested IRTs: magnitude and direction of deviation from BB reading. (a) Difference between device and subject versus subject. (b) Absolute value of difference between device and subject versus subject.

**Fig. 15:**
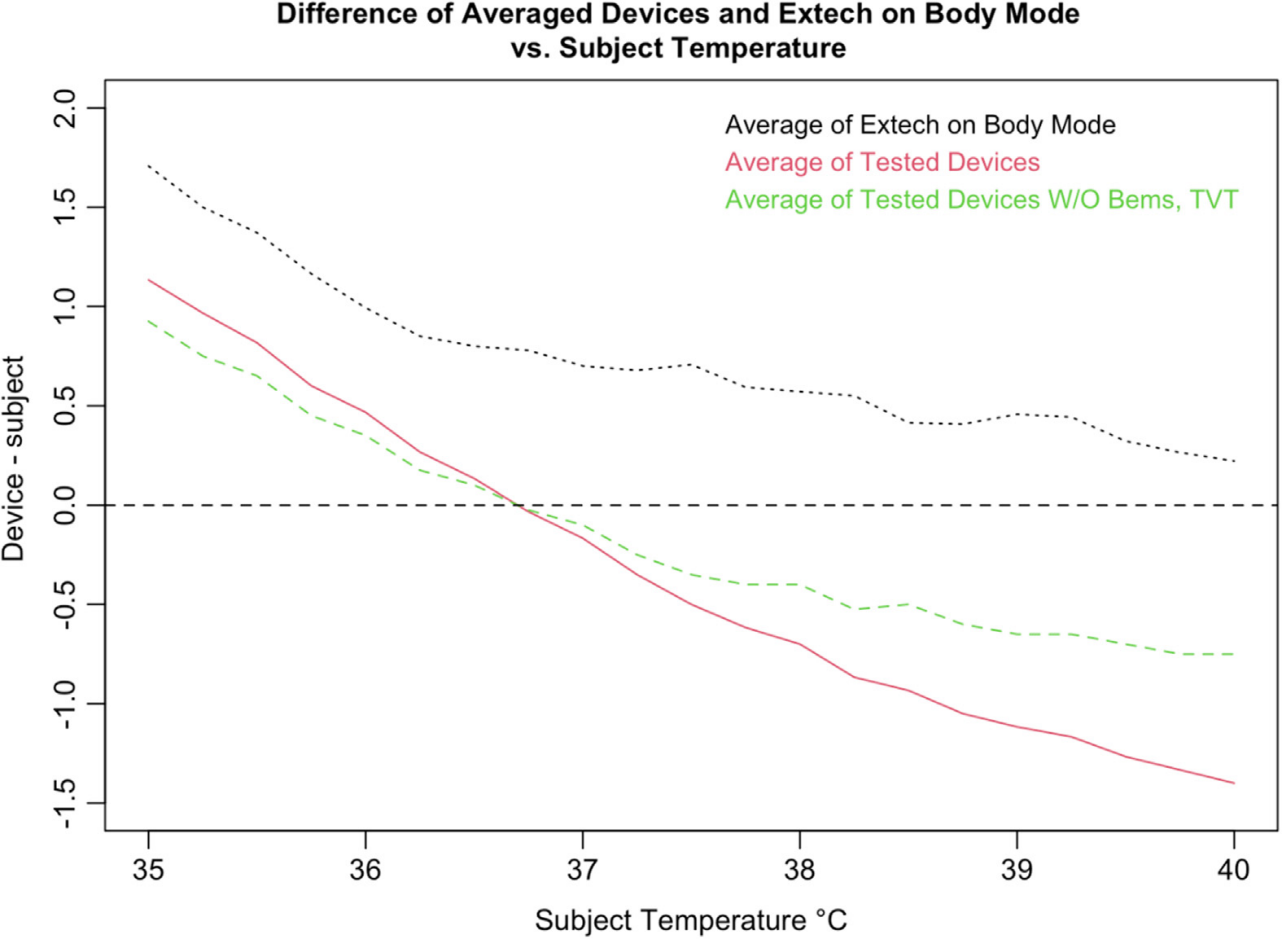
Pooled differences between tested devices and BB temperature.

An inaccurate device could have random or systematic error; however, these seven devices had deviations in readings, which suggest a systematic error that is correlated with the subject temperature, driving the readings closer to the human mean (Fig. 14). All seven tested devices systemically overestimated low temperatures and underestimated high temperatures. The error is highly correlated with temperature in a linear relation, demonstrating a clear pattern of correction to the human mean (Fig. 15).

**Fig. 16:**
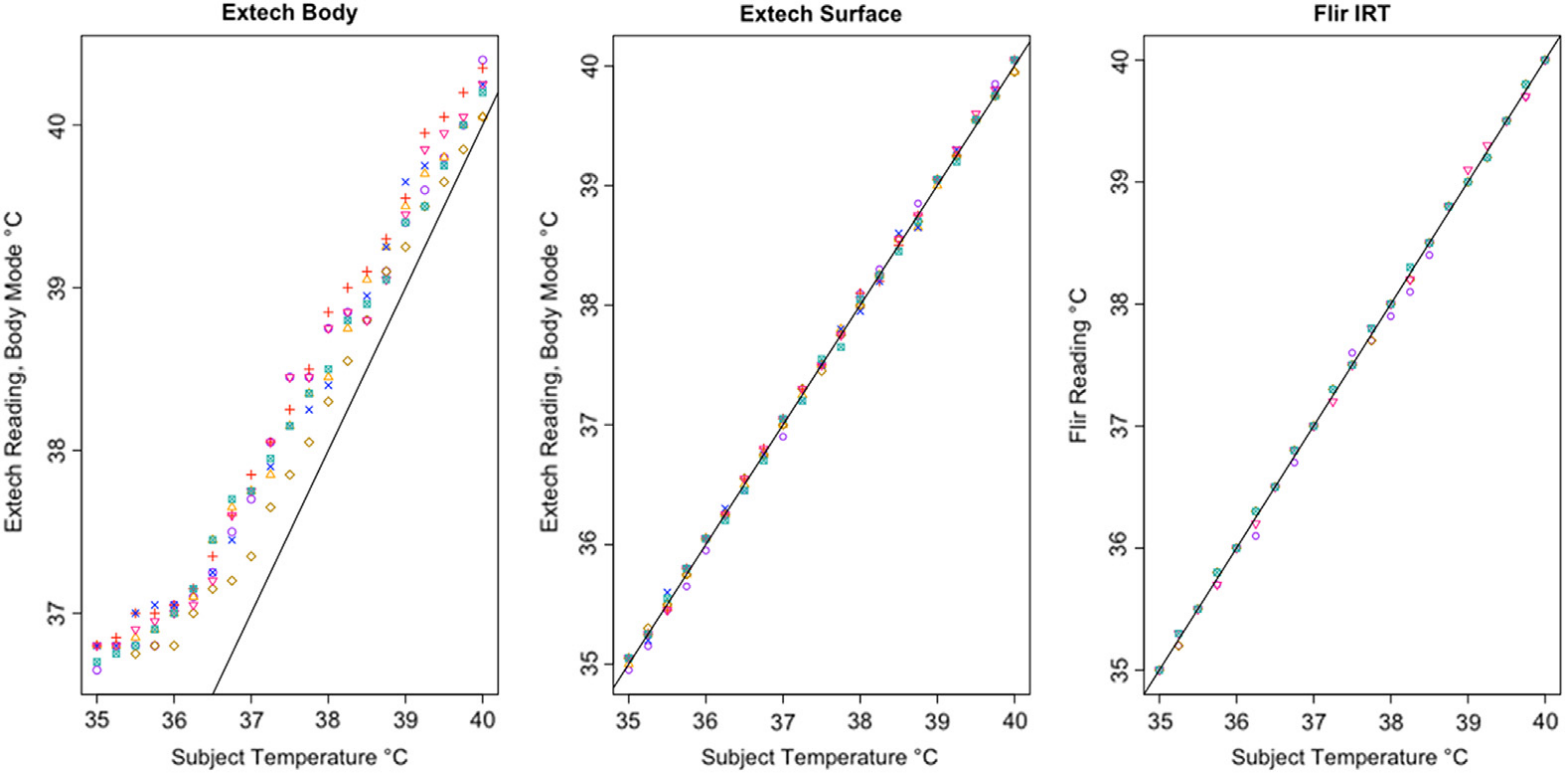
Results from control devices, FLIR E54-EST and Extech IR200.

The tested IRTs exhibited much smaller variances than the subject and the 510(k)-cleared control devices, another sign of normalization. An accurate device would be expected to demonstrate variance comparable to that of the subject, whereas a device that adjusted results toward the mean would have lower variance. In our results, the tested devices demonstrated very little variance that was nonuniformly distributed across the measured temperatures, resulting in a normalization to the human mean (Table 3). The least reliable device (Bems) consistently recorded temperature near the human mean, regardless of the BB setting, resulting in a low variance, 0.005°C. In contrast, the FDA-cleared devices had variances similar to the subject (2.4°C); the bullet cameras had notably lower variances (1.4°C to 1.6°C) and the temperature tablets had significantly lower variances (0.005°C to 0.95°C) (Table 3). The IRTs had means between 36.7°C and 37.8°C, with all devices except the Meridian having a lower mean than the BB. This analysis of variance further suggests a normalization toward the human mean in the seven tested IRTs.

**Table 3:** Variance across measured temperatures by control and tested devices.

Device	Variance (°C)	Mean (°C)	Range (°C)
Hypothetical data			
BB	2.4	37.5	35 to 40
Perfect device with no offset	2.4	37.5	35 to 40
Perfect device with a fixed offset of +1	2.4	38.5	36 to 41
An inaccurate device that does not normalize^a^	3.3	37.5	34.4 to 40.7
Experimental data			
FLIR	2.4	37.5	35 to 40
Extech surface mode	2.4	37.5	35.05 to 40.05
Extech body mode	1.3	38.2	36.7 to 40.2
Hikvision^b^	1.6	37.4	35.6 to 39.6
Dahua^c^	1.4	37.3	35.4 to 39.4
Meridian^d^	0.95	37.8	36.9 to 39.8
ZKTeco	0.85	37.3	36.3 to 39.0
Certify	0.72	37.3	36.4 to 39.0
TVT	0.16	36.9	36.6 to 37.8
Bems	0.005	36.7	36.5 to 36.8

a Simulated using randomly distributed error with standard deviation of 1.

b Calibrated with BB reference at 37°C with a fixed offset of −1.6°C as per manufacturer recommendations.

c Calibrated with BB reference at 37°C with a fixed offset of −1.6°C as per manufacturer recommendations.

d Calibrated with BB reference at 37°C with a fixed offset of +0.2°C as per manufacturer recommendations.

**Table 4:** Laboratory humidity and temperature conditions in each trial by device.

Device tested in trial	Minimum temperature (°C)	Maximum temperature (°C)	Minimum humidity (%)	Maximum humidity (%)
Hikvision	19.9	20.5	53	57
Dahua	20.4	20.7	65	67
Meridian	20.3	20.5	56	57
ZKTeco	19.8	20.2	56	58
Certify	20.2	20.5	64	67
TVT	21.8	22.4	66	70
Bems	22.2	22.6	60	65

* All devices were within manufacturer recommended temperature and humidity ranges.

## 4 Discussion

The tested devices consistently adjusted their readings relative to the subject’s temperature setting. The variation was positive at lower temperatures (hypothermic range) and negative at higher temperatures (febrile range). In other words, the tested devices measured an output that was higher than the subject at 35°C to 37°C, and lower than the subject at 37.5°C to 40.0°C, thus appearing to normalize results to the human mean. The amount of adjustment varied across the seven devices tested; the temperature tablets, as a group, showed significantly more bias than the bullet cameras.

Since surface temperatures are lower than core temperature, any offset should have increased rather than lowered temperature. At the low end of our range, the distortion was positive, closer to the mean core body temperature. However, in the febrile range the distortion was negative: the devices displayed temperatures consistently lower than the subject surface temperature. This result represents a febrile subject (a surface temperature of 37°C representing a core body temperature of 38°C) being read between 36.7°C and 36.8°C, well within the normal range. These results occurred even in a highly controlled laboratory setting with an unrealistically uniform and perfectly still subject in an unobstructed screening scenario. The Bland-Altman plots and difference plots demonstrate strong evidence of proportional bias (adjusted-R-squared values above 0.73 and Beta values below −0.208).

The error is systematic, not random, and evidenced across all seven tested devices. Experimental error can be ruled out given the high accuracy and round-to-round consistency of the 510(k)-cleared devices that were used as controls and consistent across every trial (Fig. 16). To ensure the appropriateness of the baseline measurements, the control devices (FLIR and Extech) were used in each round of testing across all 21 temperature readings. Thus, every measurement for the tested devices had a precise pairing with the control devices. Our method was conducted in accordance with FDA/IEC operational guidelines, eliminating various possible systematic effects related to screening protocol or environmental conditions. The tested devices exhibited similar accommodation to the human mean to varying degrees, which suggested that the normalization is nonrandom and not due to experimental error. The similarities in some of the curves further rule out any explanation by experimental error [see Fig. 14(a)].

**Fig. 17:**
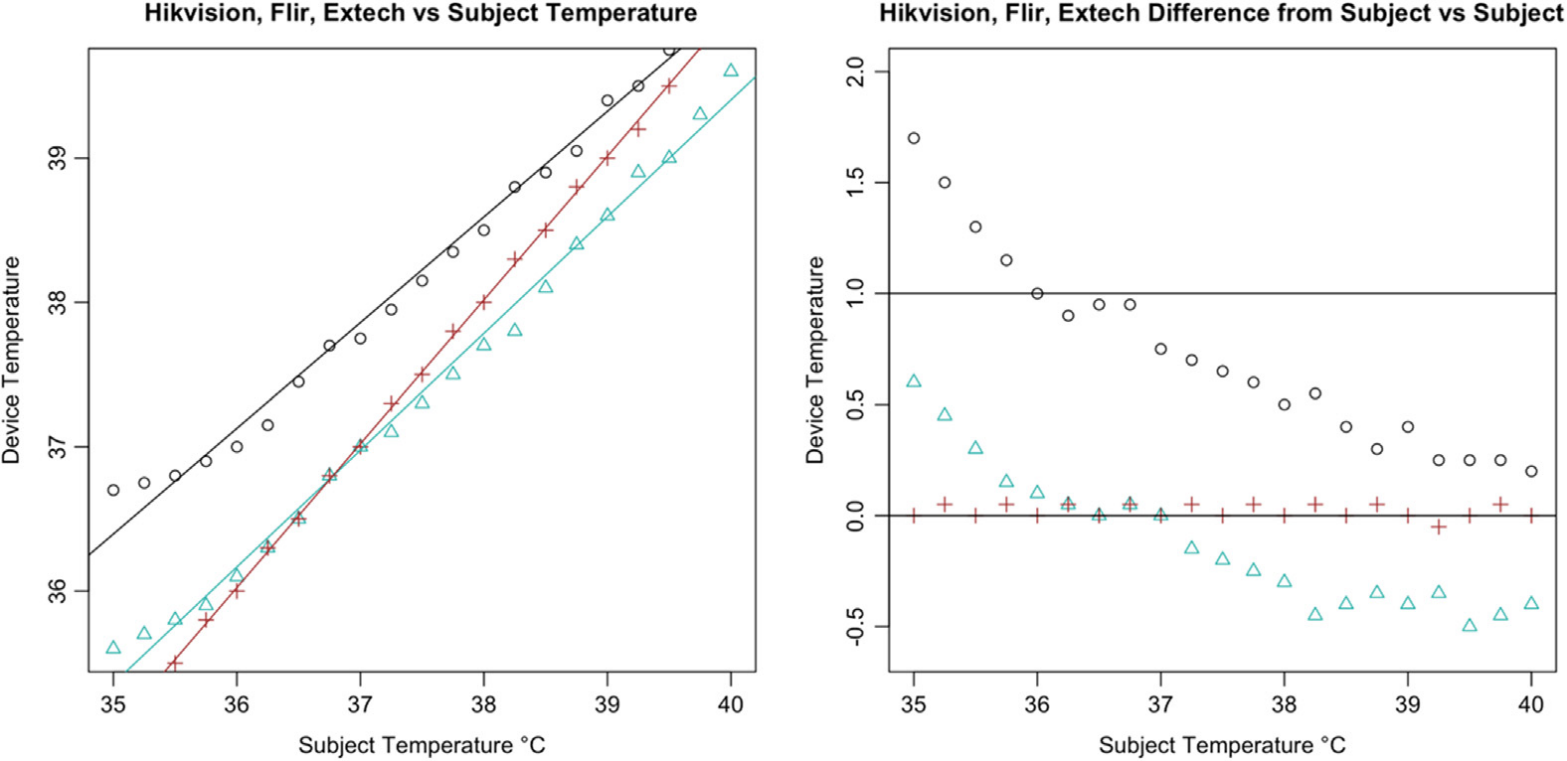
Results from Hikvision DS-2TD2636B-13/P.

We considered that the adjustment process could represent estimation of core body temperature, as with the Extech NCIT on body mode [Fig. 14(a)]. As discussed previously (1.4), body temperature is higher than surface temperature. The Extech provided readings in line with this expectation, generating estimates of body temperature that were higher than surface temperature for all readings. This was demonstrated by the Extech body curve in Fig. 17, in which adjustment is positive for all subject temperatures. Conversely, as subject temperature increased above ∼37°C, the adjustment was increasingly negative. This negative adjustment, specifically, is a key differentiator with devices that estimate body temperature, suggesting that the IRTs do not adjust readings for the purpose of estimating body temperature.

**Fig. 18:**
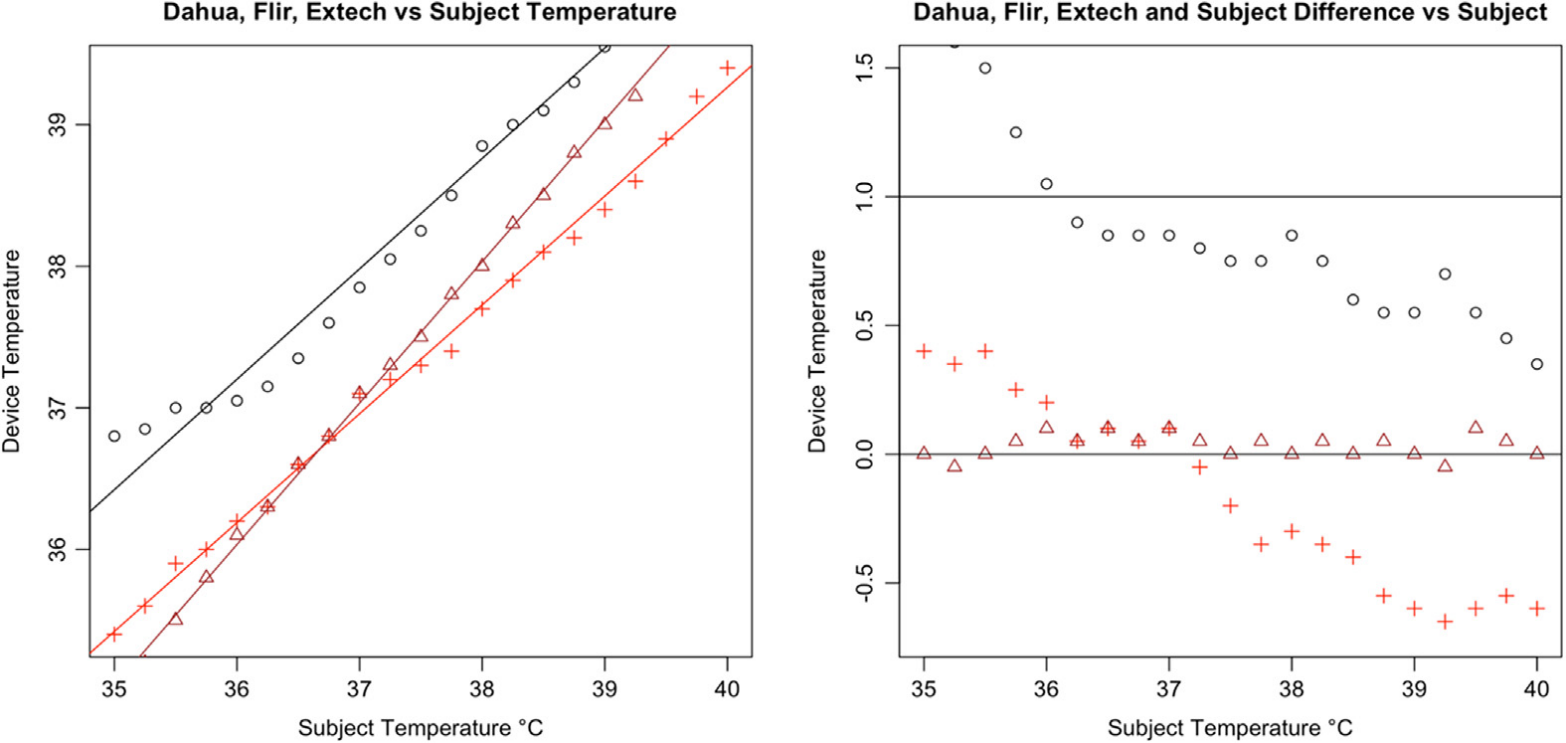
Results from Dahua DH-TPC-BF5421-T.

**Fig. 19:**
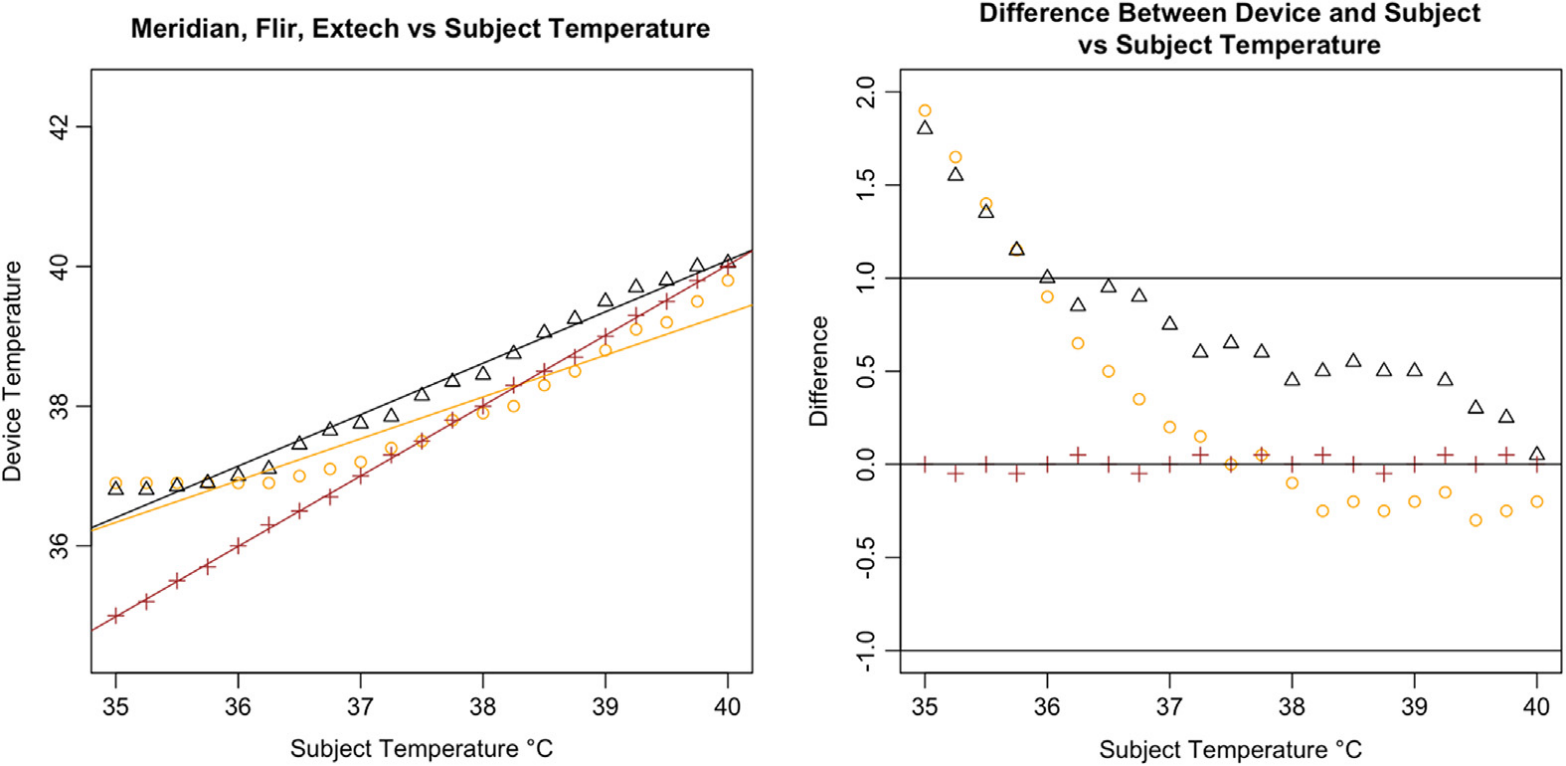
Results from Meridian Clear 2.

**Fig. 20:**
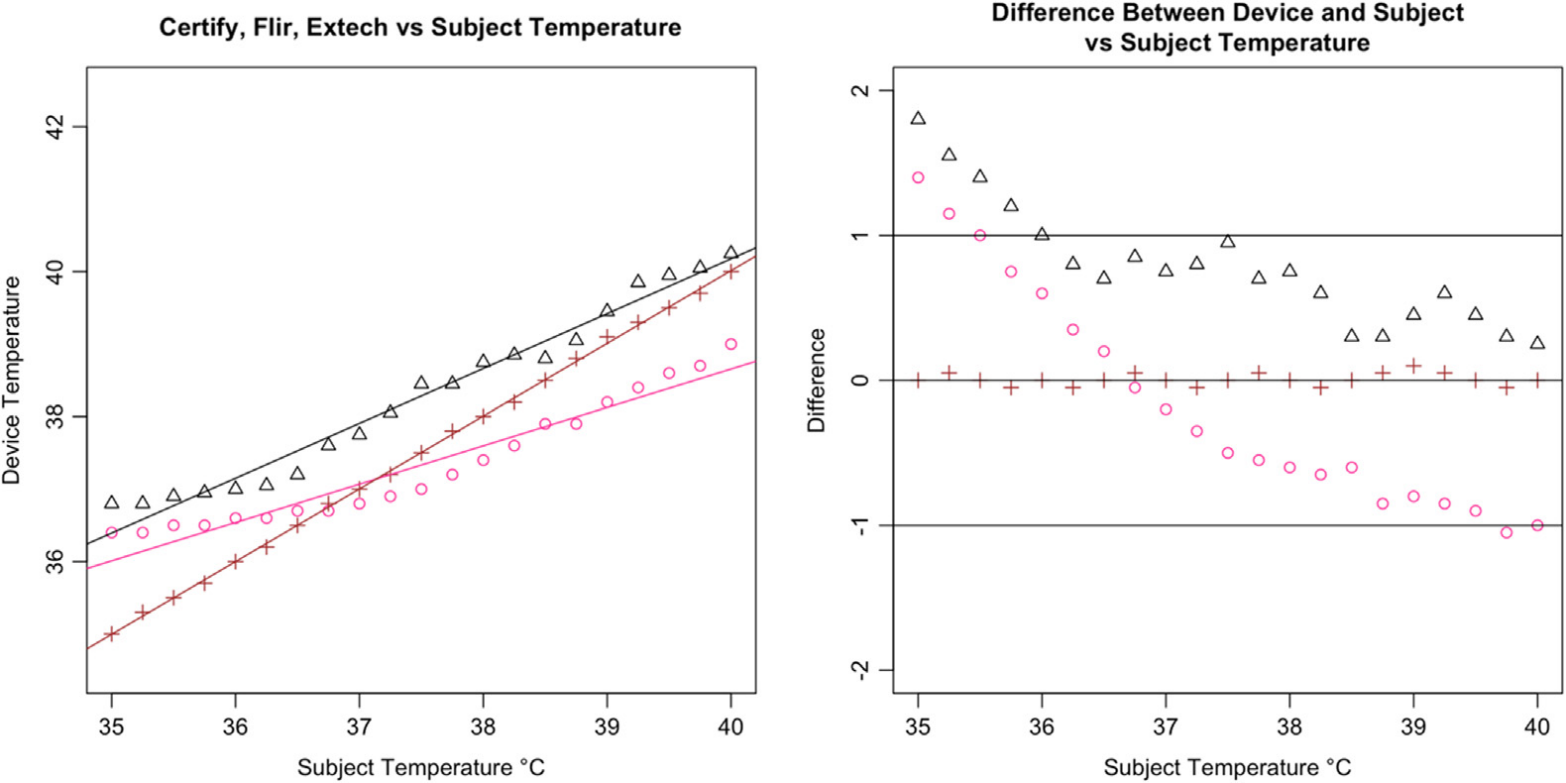
Results from Certify SnapXT Pro.

**Fig. 21:**
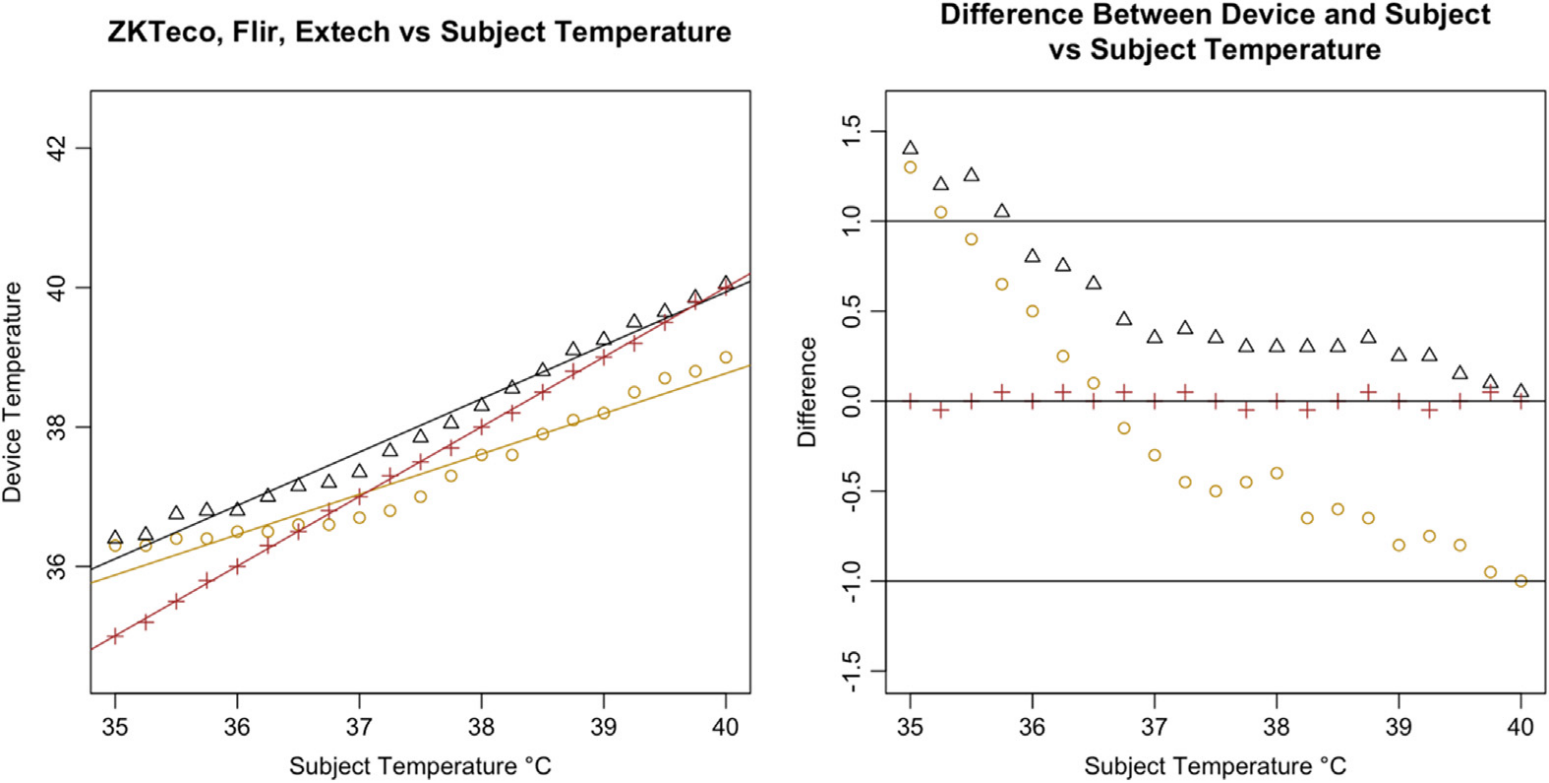
Results from ZKTeco SF1008+.

**Fig. 22:**
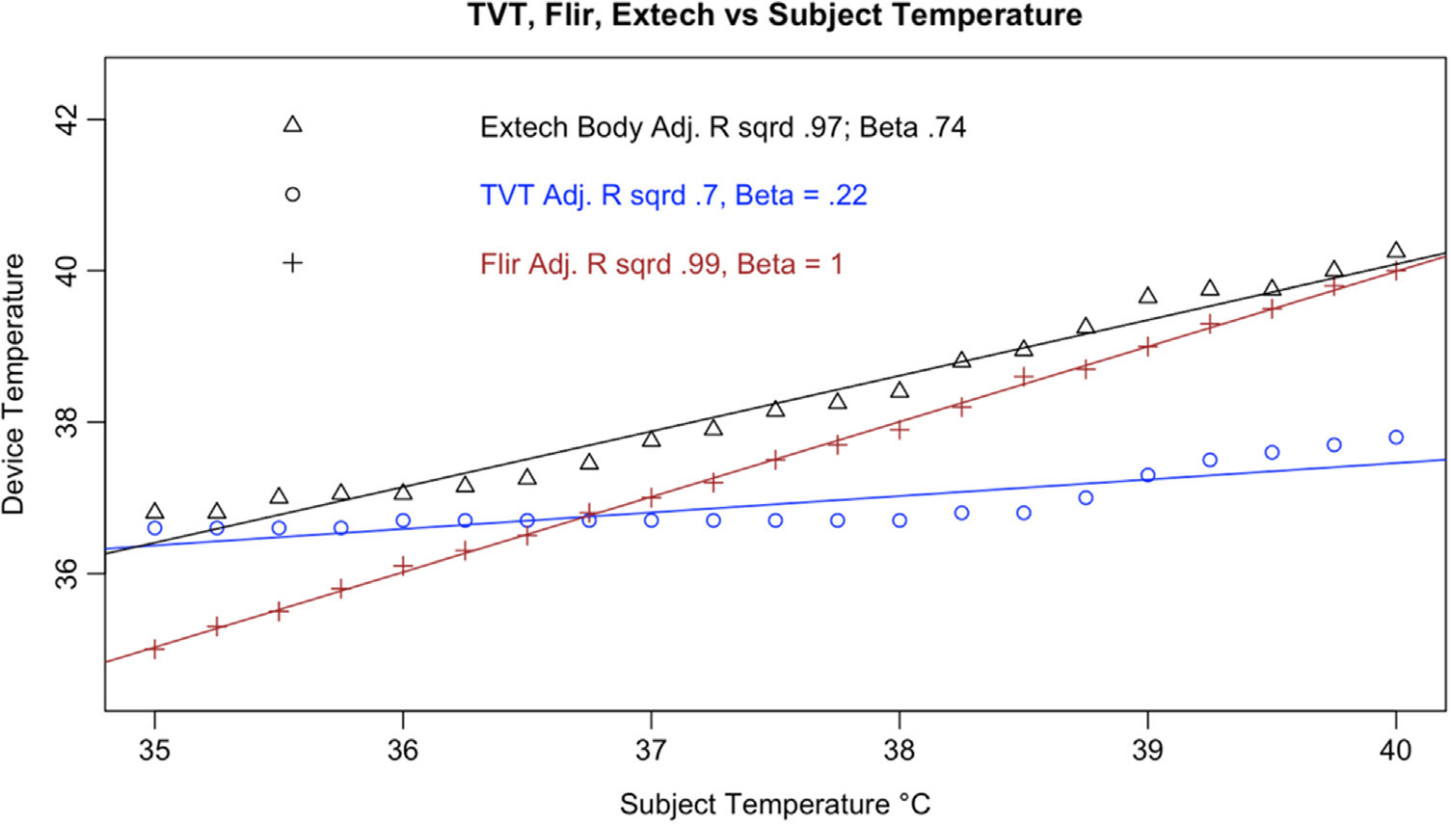
Results from TVT TD-E2128-TM.

**Fig. 23:**
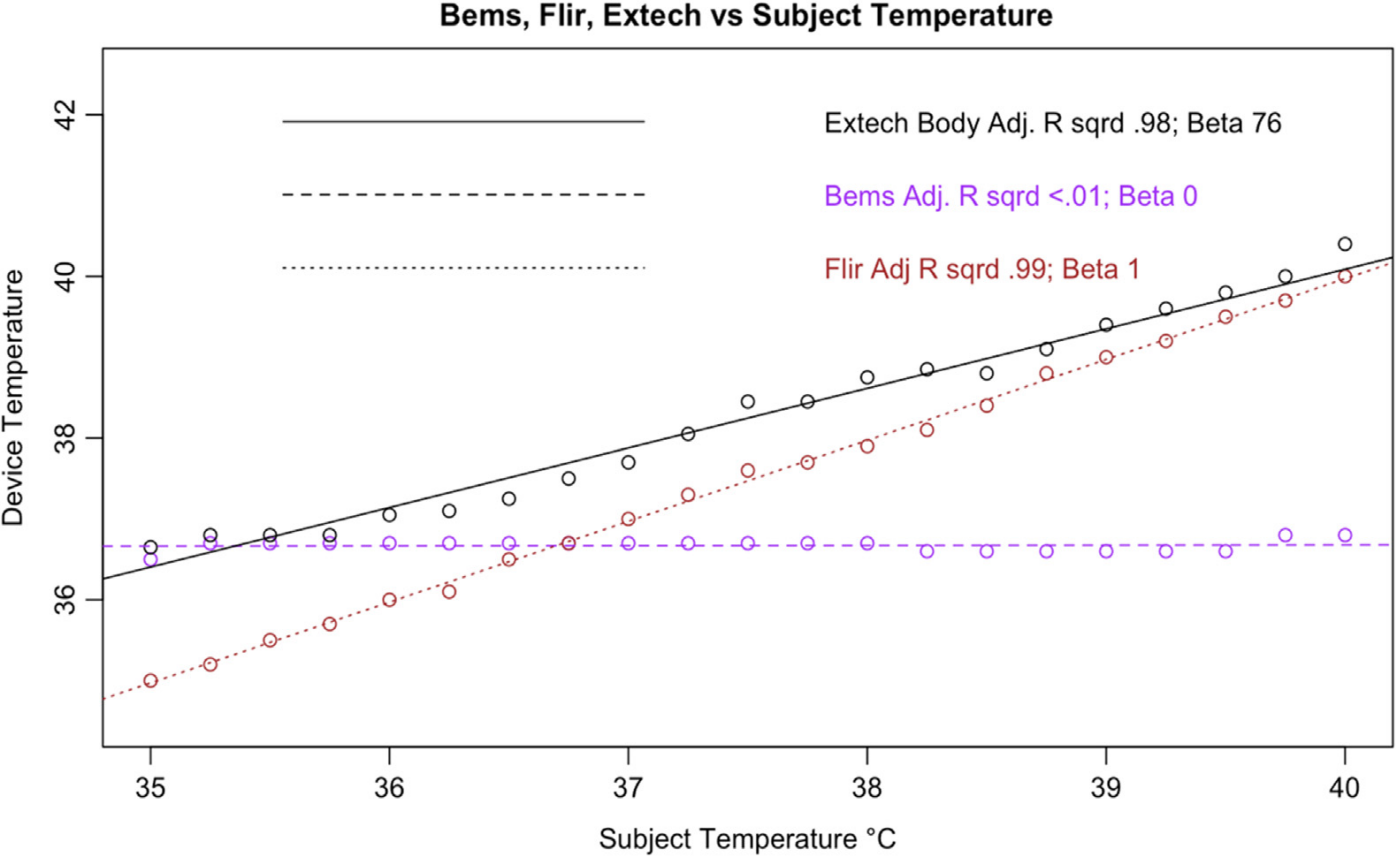
Results from Bems.

In quantitative terms, the Extech (Body) had a much stronger correlation with the subject temperature than any of the tablets we tested and had error that was less correlated with temperature than the tested devices. Although the difference was not random, it was not as linearly correlated with increasing subject temperature. While the bullet IRTs had coefficients that were more similar to the Extech, they had error values that were more correlated with subject temperature. In addition, the NCIT never provided a reading lower than the subject temperature. In contrast, as the tested IRTs approached febrile temperatures, the adjustments crossed the y-intercept and became negative (Figs. 17–23). Taken together, these findings suggest a human-target-specific algorithm that adjusts the recorded temperature to the human mean, with the strongest corrections applied to the most febrile readings. Thus, a truly febrile subject would rarely be measured as such.

### 4.1 Comparison to Previous Research

We are unaware of any papers analyzing new IRT devices for signs of normalization. This could simply be that researchers use FDA (or international equivalent) cleared devices or other purportedly accurate devices, whereas we focused on popular consumer grade devices. The tested devices were all recently marketed in 2020; if normalization in IRTs is a recent phenomenon, this may also explain why normalization has not been observed in prior research. There has been extensive research into the accuracy of NCITs and IRTs as a tool for core body estimation and for screening devices for fever or influenza like illnesses. In one recent meta-analysis of thermography sensitivity and specificity, the authors

found good pooled sensitivity and specificity for IRTs and NCITs, in excess of 0.8 and 0.9, respectively.^10^ However, these studies have largely focused on analyzing whether IRTs or NCITs could be used for accurate estimation of core temperature for clinical purposes, and therefore the selection of tested devices may have been different than our review of newly released thermography screeners. One study analyzing several NCIT devices found that the accuracy of two of the three devices tested fell outside the manufacturer specified range.^26^

### 4.2 Limitations

In our test environment, temperature conditions were aligned with manufacturer and FDA recommendations, but humidity was aligned only with manufacturer recommendations. Our ambient humidity ranges were in excess of 53% (no greater than 70%) for all tests, whereas the FDA-recommended humidity range is 10% to 50% (Table 4). Given that the test environment was uniform across all devices and within manufacturer recommendations, it is unlikely that our results were biased to reflect poorly on the tested devices and more highly on the control devices. In addition, the Extech on surface mode and FLIR IRT were accurate across every round of testing, which indicated ambient conditions did not have an impact on either device. Further, it should be expected that real-world scenarios would include high-humidity conditions, thus making it important that testing occur uniformly across all devices but in close approximation to real-world settings.

In addition, we selected one IRT in each of the seven series; follow-on research studies should consider testing multiple IRTs to detect within-device variation in the same series. This was beyond the scope of our budget, and we reflect real-world scenarios in which facilities or individuals are limited to the devices on the market and cannot purchase multiple devices for testing prior to use.

We did not use human subjects. This was considered impractical given the ongoing pandemic of communicable disease and because a properly controlled study with human subjects is more experimentally complicated. Future research could assess these devices’ performance on humans; however, it may not provide any benefit vis-à-vis our goal of assessing compensating algorithms, which is assisted by artificially controlled conditions as in our methods.

## 5 Conclusions

Our findings suggested that a nonrandom deviation from subject’s temperature was observed for all seven tested devices, yielding a distorted reading that normalized the output to the human mean. Elevated temperatures were adjusted negatively, and low temperatures were adjusted positively across all seven devices tested. In contrast, the FDA 510(k)-cleared devices demonstrated no such aberration. Further research is required to elucidate the adjustment algorithm and the underlying intention. Nonetheless, our data strongly suggested that the seven tested devices employed a nonrandom intentional modification of device readings to approximate the human temperature mean, thus rendering their effectiveness as febrility screeners uncertain.

## Code and Data

For access to our code (r) and data, e-mail chealy@ipvm.com.
